# amyloid-predict and LLPS-predict: Predicting phase separation propensities in the intrinsically disordered proteome

**DOI:** 10.1073/pnas.2531932123

**Published:** 2026-05-26

**Authors:** Samuel Lobo, Leif Griem, M. Scott Shell, Joan-Emma Shea

**Affiliations:** ^a^ Department of Chemical Engineering, University of California, Santa Barbara, CA 93106; ^b^ Department of Chemistry and Biochemistry, University of California, Santa Barbara, CA 93106; ^c^ Department of Physics, University of California, Santa Barbara, CA 93106

**Keywords:** amyloids, liquid–liquid phase separation, intrinsically disordered proteins, protein language models, neurodegenerative disease

## Abstract

Amyloid formation and liquid–liquid phase separation (LLPS) are two important phenomena in cellular biology, linked to both normal physiological functions and various pathologies. Here, we present a computational framework that scores amyloid propensities (amyloid-predict) or LLPS propensities (LLPS-predict) from protein language model embeddings, enabling rapid proteome-wide annotation of peptides and residues. amyloid-predict achieves classification performance that exceeds existing AI and physics-based tools on a hexapeptide benchmark while enabling substantially faster high-throughput screening; notably, amyloid-predict is sensitive to subtle mutational effects and is influenced by sequence patterning and context rather than amino acid composition alone. We apply these protein language model classifiers to all the IDRs in the human proteome and uncover several protein categories with significant enhancement in amyloid and/or LLPS propensity, suggesting insights into the biological roles of these protein categories. For example, signaling receptors, carbohydrate-binding proteins, and Ca^2+^ binding proteins are enriched in aggregation propensity, while mRNA-binding proteins, ribonucleoprotein complex, and nuclear matrix proteins are enriched in LLPS propensity. Interestingly, we observe patterns of both high amyloid and LLPS propensity in several amyloid-forming and prionic proteins. Together, these results provide side-by-side landscapes of LLPS and amyloid potential across the disordered human proteome while offering a rapid screening tool for basic biology, disease-mechanism studies, and rational design of peptide therapeutics.

## Significance

Proteins undergo two fundamental types of phase transitions—amyloid aggregation and liquid–liquid phase separation (LLPS)—that shape cellular organization and contribute to diseases ranging from Alzheimer’s to cancer. While these processes are central to biology, their prediction from sequence remains limited by slow or narrowly trained models. We present two fast, accurate open-source classifiers, amyloid-predict and LLPS-predict, that leverage protein language model embeddings to score phase-transition propensities across entire proteomes within hours. Applying them to all intrinsically disordered regions in the human proteome reveals protein categories enriched for amyloid or LLPS potential, including receptors, nucleic-acid-binding proteins, and prions. These findings provide a framework for rapid hypothesis generation in disease mechanisms, synthetic biology, and rational peptide design.

Intrinsically disordered proteins (IDPs) lack a fixed 3D structure (1, 2), and an estimated ~70% of proteins have intrinsically disordered regions (IDRs) (3), with approximately 35% of the human proteome consisting of disordered residues (4). IDRs have important roles in the cell, including cell recognition and signal transduction (5, 6), molecular scaffolding (7), and protein quality control (8). However, their structural heterogeneity can also predispose them to misfold and aggregate into amyloids, and this pathological aggregation occurs in several diseases, most notably in neurodegenerative diseases like Alzheimer’s, Parkinson’s, and amyotrophic lateral sclerosis (ALS) (9–11). In contrast to pathological aggregation, some IDRs form functional amyloids that play essential roles in processes such as hormone storage (12), biofilm formation (13, 14), and signaling (15, 16). Amyloid structures can be evolutionarily advantageous and amyloid formation is often highly regulated (17, 18).

Beyond amyloid aggregation, IDRs frequently undergo liquid–liquid phase separation (LLPS), a process that drives the spontaneous organization of biomolecules into dynamic, condensed phases (19). LLPS enables compartmentalization and regulation of cellular activities; notably LLPS leads to the formation of membraneless organelles such as nucleoli (20), P-bodies (21), stress granules (22), and synaptic densities (23). Current efforts also hypothesize that LLPS can promote amyloid aggregation by inducing high local concentrations of aggregation-prone protein regions (22, 24–27). Both LLPS and amyloid formation highlight the dynamic nature of IDRs and their physiological and pathological roles.

The prediction of amyloid and LLPS tendencies has thus been a major effort in the field over the past two decades. Starting with amyloids, many prediction algorithms have been developed, including Waltz (28), TANGO (29), PASTA (30), CORDAX (31), and AGGRESCAN (32), along with many meta-predictors like MetAMyl (33) and AMylPred2 (34) that leverage multiple algorithms. Broadly, these approaches have leveraged statistics from experimental amyloid datasets (Waltz, AGGRESCAN), or modeled peptide conformation, microcrystalline structure, and secondary structure with physics-based or statistical techniques (CORDAX, PASTA, TANGO). For example, CORDAX measures energies of hexapeptides by modeling side chains on available amyloid template structures using the FoldX (35) force field. Training datasets for these algorithms use varied criteria. For example, the WALTZdb hexapeptide dataset (28) uses electron microscopy and Thioflavin-T (Th-T) fluorescence assays to identify amyloids, while the TANGO dataset (29) of 10aa peptides from human proteins uses circular dichroism (CD). Moreover, Louros, et al. recently published a dataset of 15aa tau fragments that were identified as amyloids via Th-T and pFTAA fluorescence assays (36). Thompson, et al. recently published a dataset of more than 100,000 random peptides with ≤20 aa with a corresponding classifier named CANYA (37).

In this work, we first seek to develop amyloid prediction models that 1) are capable of rapidly scanning millions of fragments, 2) leverage the rich evolutionary information that has empowered modern protein AI tools like ESM (38–40), and 3) identify amyloids of different lengths and different amyloid identification criteria. Many of the physics-based amyloid prediction algorithms suffer from long inference times, do not leverage the capabilities of modern GPUs, and/or are inconvenient to deploy at scale. Protein language models (pLMs) have emerged as powerful tools to distill evolutionary information and quantify complex amino acid patterns (39) which may enable predictive models of amyloid aggregation. Recently pLMs and other machine learning methods have become more popular for studying disordered proteins (41), including for amyloid prediction such CANYA (37) and ML4MD (42). pLMs’ embeddings are more sophisticated in capturing sequence patterning than the position-specific scoring matrices of amyloid algorithms like WALTZ, and the embeddings preclude the feature engineering that has been common for machine learning models historically.

We also seek to use similar prediction workflows for LLPS. Like amyloid prediction, many computational approaches have been developed to understand and predict IDP LLPS propensity. Some frameworks describe a conceptual sequence “grammar” as a determinant of phase separation. For example, the “stickers and spacers” model emphasizes how certain residues—especially aromatic and charged residues—are “stickers” that drive multivalent interactions, while “spacer” regions—typically rich in glycines and prolines—modulate the interactions and control condensate formation (43–48). Sequence-encoded charge patterning and aromatic content are a primary determinants of IDR phase behavior, as captured by hydrophobicity scales and coarse-grained models (49–52); analytical theories such as random phase approximation (RPA) and related models provide quantitative links between sequence features and LLPS propensities (53). Early biophysics-inspired approaches such as catGRANULE (54), PLAAC (55), PScore (56), FuzDrop (57), FuzPred (58), and ParSe v1 (59) employed physicochemical heuristics—e.g., prion-like domain content, π–π interactions, and conformational entropy—to classify phase-separating proteins. Subsequently, machine-learning tools like MaGS (60), catGRANULE 2.0 ROBOT (61), PhAsEPred (62), PSPHunter (63), PSAP (64), and ParSe v2 (65) combined these engineered features with large, curated datasets. Other methods, like DeePhase (66) introduced word2vec-derived embeddings to capture “molecular grammar” that distinguishes condensation-prone IDRs from structured or non-LLPS sequences, while PICNIC (67) and Phaseek (68) expanded these concepts to incorporate structural predictions and compositional features.

Protein language models appear particularly well-suited to identify the molecular grammar that underlies LLPS formation, and can identify patterns that humans cannot easily intuit. Transformer-based models, such as ESM2 (40), show significant promise in modeling IDRs for LLPS, especially considering the recent work called ProtGPS by Kilgore, et al. who used ESM2 embeddings to predict a protein’s condensate localization (69), and LLPSWise by Pu, et al. who applied an ESM2 embedding-based model to the dataset from DeePhase to predict phase separation propensities and networks (70). pLMs are especially useful for modeling IDRs considering that multiple sequence alignments (MSA) have limited utility for disordered proteins (71). pLM-based LLPS predictors can complement other simulation-based LLPS modeling approaches like FINCHES (72) and the work by von Bülow, et al. (73) that used CALVADOS (74) simulations and machine learning to predict free energy and saturation concentration for phase separation.

Here, we specifically develop a LLPS prediction model using pLMs that is capable of rapidly and cheaply scanning millions of fragments, and then quantify the propensity of intrinsically disordered regions (IDRs) in the human proteome to drive LLPS. The pLM-based classification models developed here—called amyloid-predict and LLPS-predict—are capable of rapid inference on a GPU (e.g., dozens of predictions per second on a consumer RTX 3090 GPU). These models leverage embeddings from ESM2, which employs the transformer architecture with rich representations of amino acids.

To demonstrate the overall approach to these workflows, we apply our amyloid and LLPS predictors to all IDRs in the human proteome. Tesei, et al. (75) recently identified about ~28 k such **IDR**s—referred to as the IDRome —based on AlphaFold structural confidence scores (i.e. pLDDT). We model the amyloid and LLPS propensity of fragments along an IDR and derive a per-residue score from the fragment scores to observe general trends in amyloidogenicity and LLPS propensity of the IDR’s domains. We then analyze the IDRs’ gene ontology (GO) categories and observe that certain cellular localizations and molecular functions have significantly higher amyloidogenicity and LLPS-propensity than the remainder of the IDRome. Finally, we observe how benign and pathogenic single nucleotide polymorphisms (SNPs) locally alter the predicted amyloid and LLPS propensities, and we visualize these propensities in several well-studied amyloid-forming proteins and prions.

## Results

### amyloid-Predict and LLPS-Predict.

**Fig. 1.**
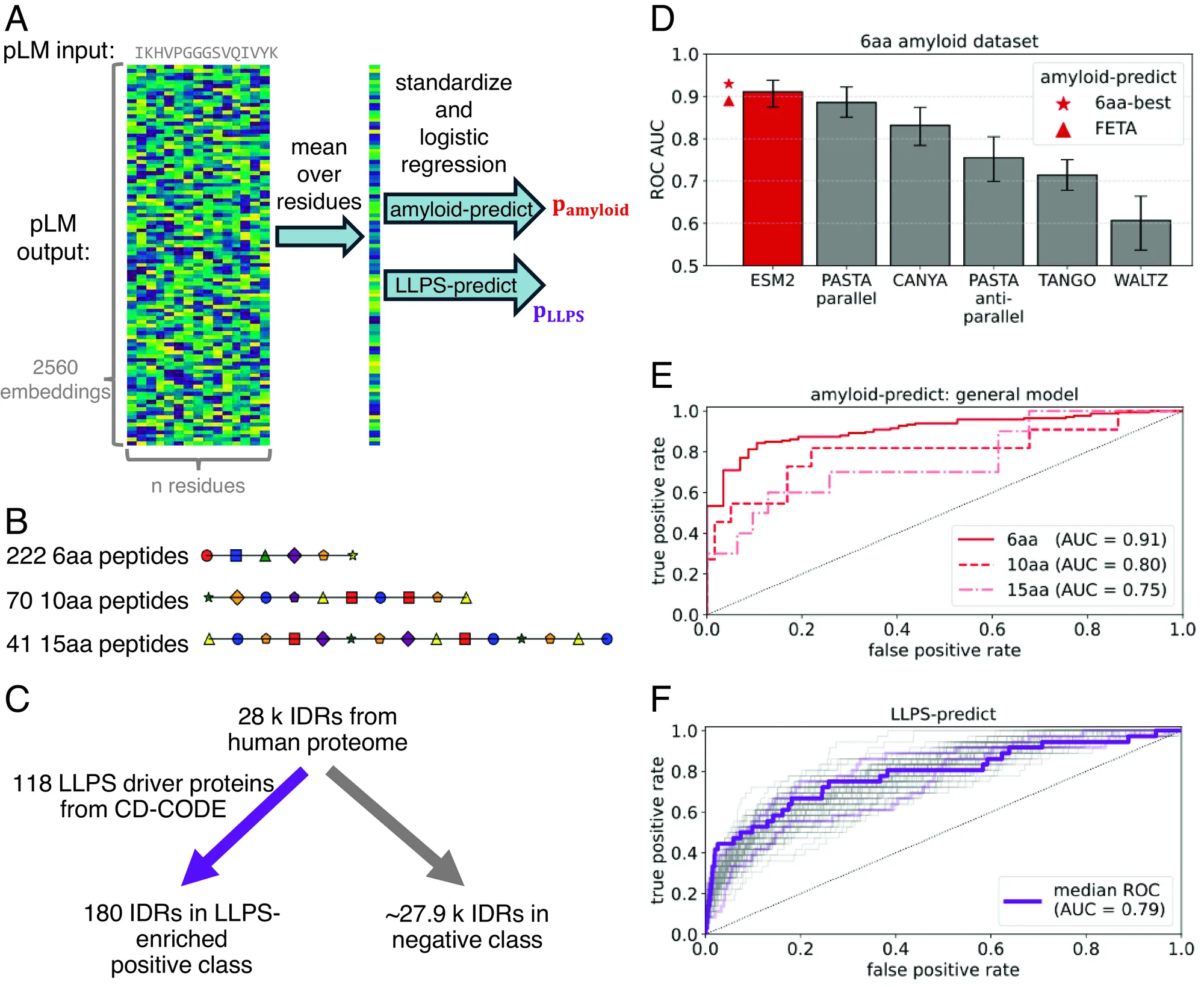
Overview of the amyloid-predict and LLPS-predict models, which use protein language model embeddings to classify peptide or IDR sequences as amyloid- or LLPS-forming. (*A*) amyloid-predict and LLPS-predict involve embedding peptide sequences with a protein language model (e.g., ESM2-3B with 2560 embeddings), mean-pooling over the amino acids, and using those as features to train a classification model to predict amyloid or LLPS formation, respectively. Note that mean-pooled embeddings reflect sequence patterning rather than only composition. (*B*) Three amyloid datasets were used to train amyloid-predict classifiers, consisting of 222 6aa, 70 10aa, and 41 15aa peptides. (*C*) LLPS-predict was trained on a dataset of 28,058 IDRs in the proteome [from Tesei et al. (75)] with a positive class of 180 LLPS-enriched IDRs based on LLPS drivers from the CD-CODE database. (*D*) Comparing ROC AUC on the hexapeptide benchmark: logistic regression model using ESM2-3B embeddings and scores from amyloid predictors in the literature (PASTA, CANYA, TANGO, WALTZ). The bar depicts the median ROC AUC from using 500 random train-test splits, and the error bars represent the 16th and 84th percentiles; the models for the ESM2 case are trained without feature selection or hyperparameter tuning. The triangle represents the ROC AUC for the amyloid-predict model called FETA (Fast ESM2-based Ten-feature Amyloid classifier) with ten selected features, and the star represents the ROC AUC for the 24-feature model. (*E*) ROC of the general amyloid-predict model, evaluated on each dataset with LOOCV. (*F*) ROC of the LLPS-predict model. This median ROC AUC curve from 100 train-test splits is highlighted. See *SI Appendix*, Appendix S1 for model training details.

We find that the rich contextual information in protein language models, pretrained on evolutionary sequences, is sufficient to predict phase changes from peptide sequences alone. We train lightweight logistic regression models on mean-pooled embeddings obtained from the ESM2-3B protein language model, as illustrated in Fig. 1*A*. These mean-pooled representations encode sequence patterning and higher-order context, not merely amino acid composition. The amyloid prediction models, called amyloid-predict, were trained on three amyloid datasets from the literature consisting of 6aa (28), 10aa (29), and 15aa (36) peptides (Fig. 1*B*). The LLPS prediction model, called LLPS-predict, was trained to distinguish IDRs from LLPS driver proteins (i.e. proteins that can induce LLPS on their own, identified by the CD-CODE (76) database) from other IDRs in the human proteome, as identified by Tesei et al (75) (Fig. 1*C*). See *SI Appendix*, Appendix S1 for model training details. On a consumer GPU (RTX 3090 Ti), inference throughput ranges from ~0.6 to ~200 sequences per second depending on batch size and peptide length (*SI Appendix*, Table S1).

We find that our 6aa amyloid classifier exceeds the classification performance with this test set of several other amyloid predictors from the literature: PASTA (30), CANYA (37), TANGO (29), WALTZ (28). Our classifier achieves a ROC AUC of 0.91 on the dataset of hexapeptides without feature selection or hyperparameter tuning, exceeding the performance of PASTA-parallel (AUC=0.89), CANYA (0.83), PASTA-antiparallel (0.76), TANGO (0.71), and WALTZ (0.61); see Fig. 1*D*. Classification performance is improved by feature selection (using L1 regularization; see *SI Appendix*, Fig. S1) and tuning L2 regularization strength with cross-validation, achieving a ROC AUC of 0.93 in a model called amyloid-predict 6aa best (red star, Fig. 1*D*). We find that just ten embeddings selected by L1 regularization were sufficient for ROC AUC of 0.89 (red triangle, Fig. 1*D*), a small feature size that may enable interpretable insight from specific embeddings with future work; we call this model FETA (Fast ESM-based Ten-feature Amyloid classifier). In throughput benchmarks (*SI Appendix*, Table S1), amyloid-predict/LLPS-predict run substantially faster than the public PASTA 2.0 web server wall-clock for matched peptide lengths and batch sizes, noting that web-server times include network and server overhead.

We then train a general amyloid aggregation classifier on the three amyloid datasets of fixed length (6aa, 10aa, 15aa), weighing each dataset evenly. The ROC AUC for the 6aa, 10aa, and 15aa datasets are 0.91, 0.80, and 0.75, respectively, when evaluating with LOOCV (Fig. 1*E*). With the broadened training set, this model is likely to be the most generalizable to new amyloid sequences, although we expect it to be less useful for longer peptides, considering that 15aa is the longest length in the training data. To assess cross-dataset generalization, we additionally performed leave-one-dataset-out (LODO) evaluation and cross-dataset transfer tests; results are summarized in *SI Appendix*, Tables S2 and S3.

Furthermore, we train models specific to the 10aa and 15aa amyloid datasets, and compare these to classifiers trained with various ESM2, ESM3, and ESMC models’ embeddings (*SI Appendix*, Figs. S2–S4). The amyloid-predict 10aa model achieved a ROC AUC of 0.85 (*SI Appendix*, Fig. S3), and the 15aa model achieved a ROC AUC of 0.86 (*SI Appendix*, Fig. S4). We did not observe obvious patterns in classification performance with model architecture or size, although additional training data and alternate training schemes may yield benefits to certain model architectures (see Discussion). We compare amyloid-predict classifiers to the recent CANYA amyloid classifier and find that amyloid-predict has notably better performance on the 10aa dataset while comparable performance in the other datasets (*SI Appendix*, Fig. S5). We additionally train a 20aa amyloid classifier with ESM2-3B embeddings on three CANYA datasets and find similar performance to the CANYA model (*SI Appendix*, Fig. S5).

We find that amyloid-predict is sensitive not only to amino acid composition but also to sequence patterning and residue context. To isolate compositional effects, we performed composition-preserving permutation analyses of well-characterized amyloidogenic peptides (*SI Appendix*, Fig. S6). For example, exhaustive permutations of the tau PHF6 hexapeptide (VQIVYK) show a broad distribution of predicted aggregation propensities despite identical amino acid composition (*SI Appendix*, Fig. S6*A*). Subclassification of these VQIVYK permutations reveals systematic positional effects: sequences in which the hydrophilic residues Q and K maintain similar positional parity (e.g. both even or both odd residue IDs) tend to retain higher predicted aggregation propensity, whereas arrangements that place these residues adjacent near the peptide center show reduced scores (*SI Appendix*, Fig. S6*B*). These trends are consistent with disruption of hydrophobic–polar spacing known to influence amyloid formation (77). Together, these controls indicate that amyloid-predict captures contextual sequence patterning beyond amino acid composition alone.

Consistent with this interpretation, the model is also sensitive to subtle mutational differences that correlate with independent biophysical measurements. Longhini et al. (78) reported on a highly amyloidogenic tau fragment called jR2R3 P301L, a moderately aggregation prone fragment called jR2R3 (without the P-to-L mutation), and an aggregation-resistant fragment called jR1R3 (with four additional mutations), and they observed that intermolecular hydrogen bonding from replica exchange molecular dynamics (REMD) simulations correlate to the aggregation propensity. We find that our general model’s predictions correlate with the REMD intermolecular hydrogen bonding propensities: the highly aggregating, moderately aggregating, and aggregation-resistant peptides have amyloid-predict logits of 7.8, 5.2, and −1.0, respectively, while their intermolecular hydrogen bond probabilities from REMD were 19%, 12%, and 3.6% (*SI Appendix*, Fig. S7). These results point to the fact that our amyloid classification model picks up biophysically meaningful properties that it was not explicitly trained on.

Next, we train our LLPS IDR classifier, called LLPS-predict, to distinguish a positive class of IDRs enriched in LLPS propensity from other IDRs in the human proteome; this model achieves a ROC AUC of 0.79 (16th–84th percentile: 0.75 to 0.81; Fig. 1*F*). Other pLMs’ embeddings yield similar classification performances (*SI Appendix*, Fig. S8). This model is catered to disordered regions of proteins, making it unique from other LLPS classifiers. While this model is subject to the biases inherent in the CD-CODE dataset (used to construct the positive class), we hypothesize that it has detected some general properties of LLPS drivers rather than merely learning similarity to well-studied LLPS-drivers. To test this, we performed leave-one-GO-molecular-function-out evaluation, withholding all IDRs annotated with each of 39 well-represented GO molecular function terms during training and evaluating on the held-out term. Performance remains substantial (median held-out ROC-AUC = 0.73; “nucleic acid binding” held out: ROC-AUC = 0.73), supporting that LLPS-predict captures transferable sequence signals beyond any single dominant functional category (*SI Appendix*, Table S4). Consistent with this, when applied to composition-matched rearrangements of a low-complexity segment from FUS, LLPS-predict tends to assign higher scores to well-spaced “sticker” patterns than to clustered arrangements (*SI Appendix*, Fig. S9), demonstrating sensitivity to sequence patterning beyond amino acid composition. Together, these results suggest LLPS-predict may be used to identify LLPS driver proteins and annotate IDRs. This version of LLPS-predict does not appear to be sensitive to nuanced mutational differences, partly due to its training on many large, variable-length IDRs; a single mutation has a weaker signal, especially when averaging the embeddings over many residues in a long IDR.

### Annotating the Human IDRome.

**Fig. 2.**
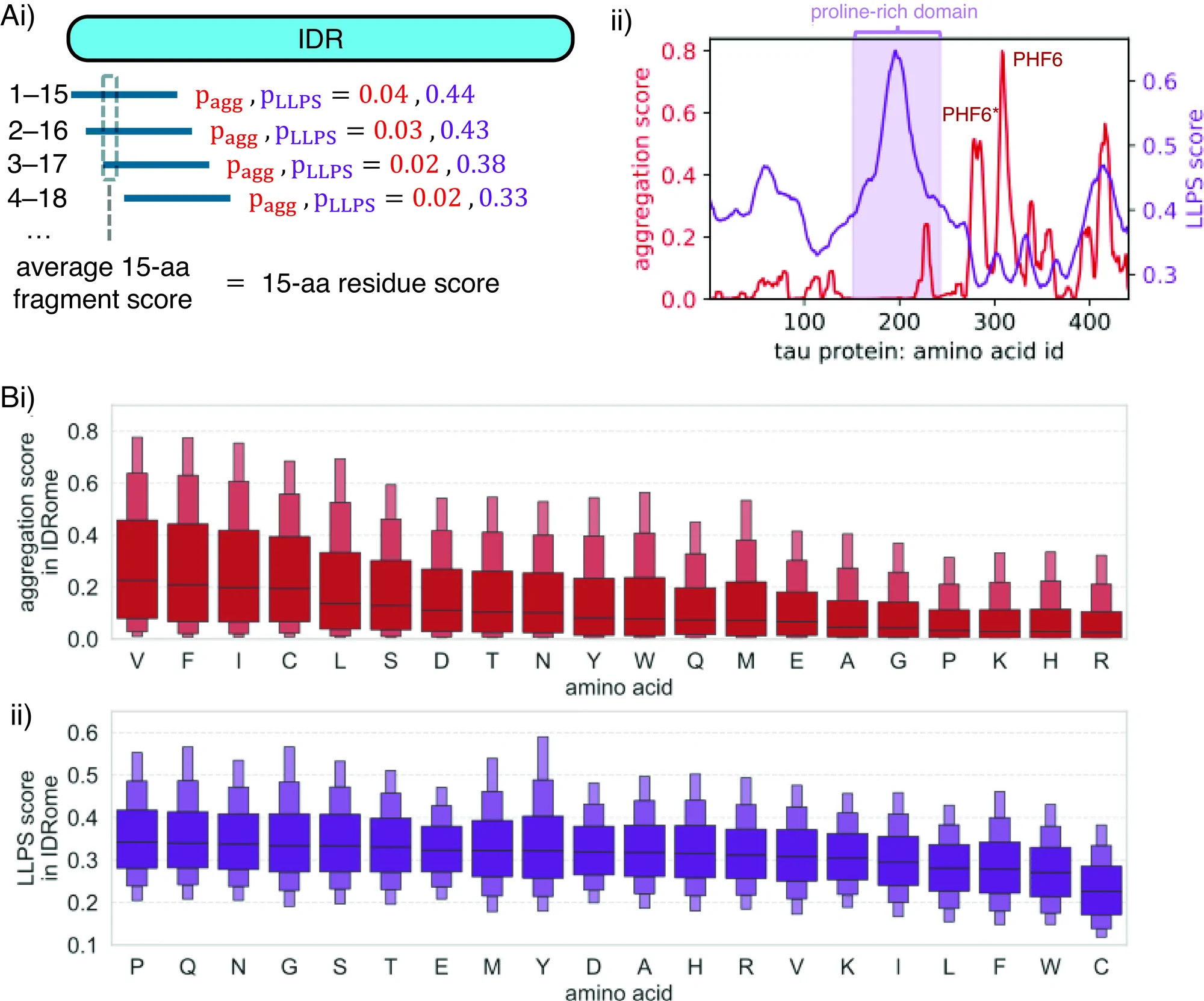
Overview of how per-residue aggregation and LLPS propensities are derived and compared across IDRs and amino acids. (*A*) (*i*) Our approach to translate fragment scores into per-residue scores; see *SI Appendix*, Appendix S2 for details. (*ii*) Per-residue aggregation scores (red) and LLPS scores (purple) for tau protein (2N4R). The proline-rich domain has high LLPS scores, while the well-studied PHF6 and PHF6* hexapeptides have high aggregation scores. (*B*) Comparing amyloid aggregation score distributions (*i*) and LLPS score distributions (*ii*) for each of the twenty amino acids across the disordered proteome (IDRome). Amino acids are ranked from *Left* to *Right* by their median score. The 3-layered boxenplot indicates, e.g., the 75% (*Top* of dark purple), 87.5% (*Top* of lighter purple), and 93.25% (*Top* of lightest purple) percentiles of the distribution.

Using the developed amyloid and LLPS prediction models, we annotate the amyloid aggregation propensity and LLPS propensity of the IDRome—the collection of IDRs in the human proteome. First, we devise a method to score the propensity for a residue to participate in amyloid-like and LLPS-like interactions. We fragment each IDR into overlapping peptides, score fragments with our classifiers, and then derive a per-residue score from the fragment scores (see Fig. 2*A*, Methods). With our general amyloid-predict classifier we predict aggregation probabilities for overlapping fragments of size six, ten, and fifteen, and we average the per-residue scores for each fragment probe size to obtain an aggregation score for each residue. Similarly, with our LLPS classifier, we calculate LLPS probabilities for overlapping fragments of size 15, 25, and 40 (*SI Appendix*, Appendix S2 and Fig. S10), and we average per-residue scores to obtain an *LLPS score* for each residue. The three fragment lengths allow the model to consider shorter- and longer-range interactions and patterns.

These per-residue scores are a convenient way to highlight regions/domains of a protein that may drive amyloid aggregation or LLPS formation. As an example, we show in Fig. 2 *A*, *ii* the predicted LLPS and aggregation scores for the tau protein, a microtubule binding protein that can form both condensate and fibrillar assemblies. Our predictions identify, in agreement with experimental observations, the PHF6 and PHF6* (79) segments of the protein as highly aggregation prone and the proline-rich domain (80, 81) as highly LLPS prone (Fig. 2 *A*, *ii*). We discuss this example deeper in the Discussion section.

After applying this dual scoring scheme to the disordered proteome, we report the amyloid aggregation score distributions and LLPS score distributions for each of the twenty amino acids in Fig. 2*B*. The highest amyloid aggregation scores are obtained by valine, phenylalanine, and isoleucine (Fig. 2 *B*, *i*). Generally, the highest LLPS scores are obtained by proline, glutamine, asparagine, glycine, and serine (Fig. 2 *B*, *ii*). Tyrosine also stands out: the top scoring 12.5% of tyrosine residues in the proteome score more highly than other amino acids (see light purple box above Y in Fig. 2 *B*, *ii*). For context, the IDRome—and especially the IDRs in LLPS drivers—has more prolines, serines, and glycines than the full human proteome (*SI Appendix*, Fig. S11).

**Fig. 3.**
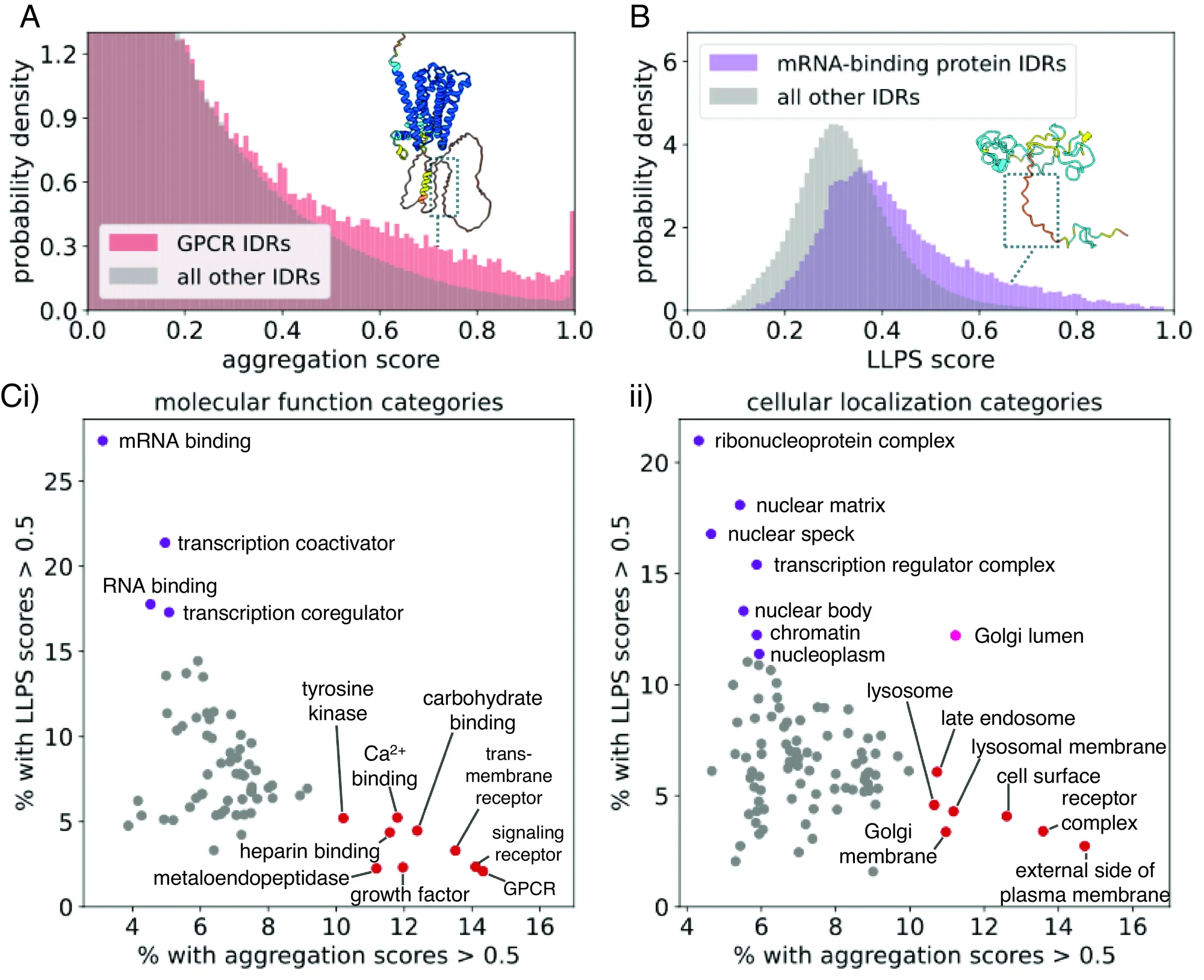
Comparison of the LLPS- and aggregation-propensities of protein categories (i.e. GO terms) from the disordered human proteome (IDRome). (*A*) GPCR IDR residues have the highest aggregation scores and (*B*) mRNA-binding IDR residues have the highest LLPS scores of all the molecular function categories. Inset in (*A*) is the M2 muscarinic receptor, which scores between 0.58 to 0.80 for residues 324 to 349. Inset in (*B*) is the CNBP mRNA-binding protein, which has scores ranging between 0.60 to 0.68 for residues 21 to 36. Inset AlphaFold structures are colored by pLDDT to highlight IDRs. (*C*) Each protein category is plotted by the percentage of residues with scores larger than 0.5. Molecular function categories (*i*) and cellular localization categories (*ii*) are plotted, and various outlier categories are annotated. Points represent residue-weighted fractions of high-scoring residues within GO categories; complementary IDR-level enrichment statistics are provided in *SI Appendix*, Table S5.

We identify significant variations in amyloid and LLPS propensities across protein categories. To quantify these trends, we evaluate the percentage of highly aggregation- and LLPS-prone residues (i.e., per-residue scores >0.5) within each Gene Ontology (GO) molecular function and cellular localization category. Overall, approximately 7.5% of all amino acids in the IDRome were predicted to be highly aggregation prone. Certain molecular function categories exhibit notably higher aggregation propensities, including G-protein-coupled receptors (GPCRs) (~14.3% of IDR residues are highly aggregation prone), signaling proteins (~13.5%), carbohydrate-binding proteins (~12.4%), calcium-binding proteins (~11.8%), and heparin-binding proteins (~11.6%) (Fig. 3 *C*, *i*). Additionally, several cellular localization categories are enriched in aggregation-prone IDRs from the residue-level analysis, with the highest percentages observed in proteins associated with the external side of the plasma membrane (~14.7%), cell surface (~12.6%), and Golgi lumen (~11.2%) (Fig. 3*C*, *ii*).

There are several reasons why amyloid-predict may assign high aggregation propensities to GPCR and other membrane-associated IDRs (Fig. 3 *A* and *C, i*). High-scoring regions likely share sequence features present in amyloid-forming training examples, such as a propensity for intermolecular hydrogen bonding and self-interaction. In membrane-associated proteins, elevated scores could reflect functional oligomerization interfaces, generic self-association motifs in signaling pathways, or sequence features correlated with membrane association (e.g., increased hydrophobicity). More generally, for membrane-associated categories these scores should be interpreted as model-rated amyloid-like interaction propensity, which may reflect aggregation-relevant sequence features and/or sequence features correlated with membrane association.

Next, we examine the per-residue LLPS propensity scores for protein categories in the human IDRome. Approximately 6.8% of all the amino acids are highly LLPS prone (i.e. with scores greater than 0.5). Several molecular function categories have substantially higher percentages of LLPS-prone IDR residues. For example, ~27.4% of mRNA-binding protein IDRs were highly LLPS-prone (Fig. 3 *C*, *i*), consistent with their roles in biomolecular condensates. Other highly enriched molecular function categories in the residue-level analysis include transcription activity coactivator proteins (~21.4%), chromatin binding proteins (~13.7%), and SH3 domain binding proteins (~11.4%); see Fig. 3 *C*, *i*. Some highly enriched cellular localization categories in the residue-level analysis include the ribonucleoprotein complex (~21.0%), the nuclear matrix (~18.1%), the kinetochore (~11.0%), and bicellular tight junction (~10.0%); see Fig. 3 *C*, *ii*.

Statistical significance of GO enrichments was evaluated using an IDR-level enrichment analysis with multiple-testing correction. Specifically, we compared the distribution of per-IDR fractions of residues exceeding the threshold score between each GO category and all remaining IDRs using the Brunner–Munzel test followed by Benjamini–Hochberg FDR correction. Results were robust to reasonable variation in the per-residue threshold (0.4 to 0.6), as summarized in *SI Appendix*, Table S5. We additionally performed regression analyses controlling for hydrophobicity and IDR length. As expected, this adjustment attenuates several weaker amyloid enrichments, but most category-level signals persist (*SI Appendix*, Table S6), consistent with the models capturing features beyond simple hydrophobicity or length.

It is important to note that the differences in aggregation and LLPS propensities predicted by our models is not simply due to compositional differences between protein categories and that our predictors account for sequence patterning. For each protein category (i.e. gene ontology molecular function and cellular localization category), we compute a composition-averaged score by multiplying the amino acid composition frequencies by the amino acid’s average score (from the amino acid score distributions in Fig. 2*B*). The averaged scores correlate poorly with the percentage of high scoring residues in molecular function categories (R^2^ = 0.50) and cellular localization categories (R^2^ = 0.39), indicating that amino acid composition explains part but not all of the aggregation propensity variation across categories (*SI Appendix*, Fig. S12). For example, heparin-binding proteins have a composition that would suggest average aggregation propensity, but amyloid-predict identifies it as one of the most aggregation-prone molecular function categories. The composition-averaged LLPS scores have higher correlations to the percentage of high scoring residues in molecular function categories (R^2^ = 0.69) and cellular localization categories (R^2^ = 0.58), suggesting that LLPS-predict is comparatively more influenced by composition than amyloid-predict (*SI Appendix*, Fig. S12).

Complementing this analysis, composition-controlled tests indicate that both predictors capture sequence patterning beyond amino acid composition alone. As noted above, permutation analyses of the tau PHF6 hexapeptide (VQIVYK) and rearrangements of FUS low-complexity segments show sensitivity to residue spacing in amyloid-predict and LLPS-predict, respectively (*SI Appendix*, Figs. S6 and S9). Furthermore, composition-preserving scrambling systematically reduces scores for both models, with a larger average decrease for amyloid-predict (*SI Appendix*, Fig. S13). Together with the composition-averaged analyses, these results suggest stronger dependence on contextual residue patterning for amyloid-predict, while LLPS-predict retains comparatively greater compositional influence.

### Visualizing Protein LLPS and Aggregation Scores.

**Fig. 4.**
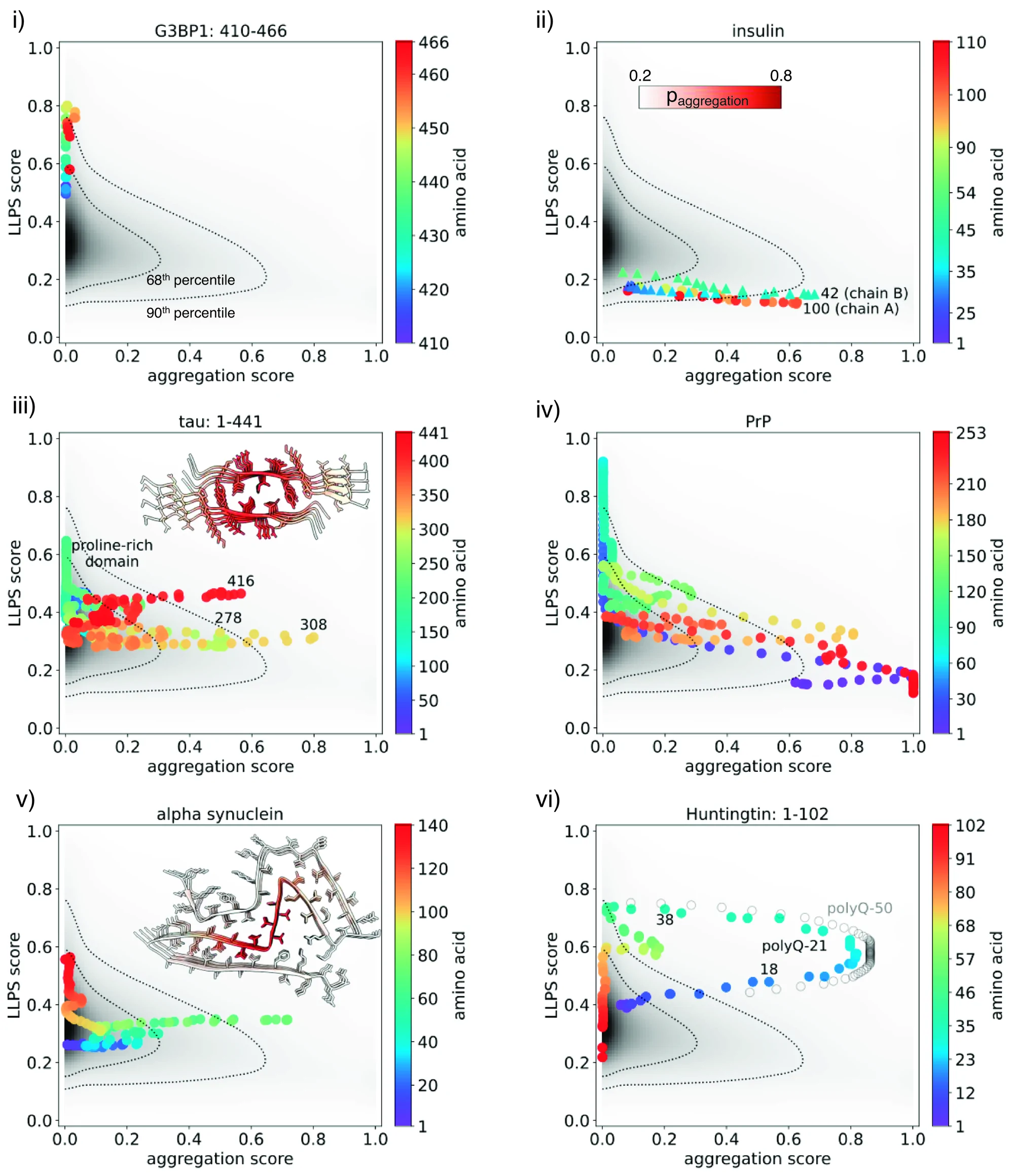
The per-residue LLPS and aggregation scores of several proteins: G3BP1 (*i*), insulin (*ii*), tau (*iii*), PrP (*iv*), alpha synuclein (*v*), and Huntingtin (*vi*). The color represents the amino acid number with the N terminus as purple and the C-terminus as red. The contours represent the 68th and 90th percentiles for all the IDR amino acids in the human proteome; the grays in the background give a heatmap of those IDR amino acids. Inset images are amyloid structures solved from NMR (*ii*, PDB #8RVT) or cryo-EM (*iii*, PDB #8PPO; *v*, PDB #8A9L); here, reds highlight residues with high aggregation propensities, the darkest red corresponding to p_agg_ of >0.8 and white corresponding to <0.2. In Huntingtin, the polyQ region from 18 to 38 is annotated (polyQ-21) and hollow circles represent residue scores when polyQ is expanded to 50 Q’s (polyQ-50) to model phase separation propensities in a diseased state.

The outputs of LLPS-predict and amyloid-predict allow us to identify per-amino acid LLPS and amyloid scores in the context of an individual protein. Fig. 2 *A*, *ii* shows one method to display these results. In Fig. 4 and *SI Appendix*, Figs. S14–S18, we present the same data in a more interpretable manner by visualizing individual proteins through each residue’s LLPS versus aggregation score. This method reveals different classes of IDRs that are prone to phase separation. A first class has high LLPS but low aggregation scores: one example is the G3BP1 C-terminal IDR (Fig. 4*i*), a multifunctional RNA-binding protein that plays a central role in stress granule formation and RNA metabolism (82), and its C-terminal arginine- and glycine-rich IDR is observed to drive phase separation (83). A second class has high amyloid aggregation propensity but low LLPS propensity: one is insulin (Fig. 4*ii*), which is seen to aggregate at the site of repeated injections in some diabetic patients (84) and has an amyloid structure solved with solid-state NMR (85) (PDB #8RVT, see Fig. 4*ii*, *Inset*). Other hormones like glucagon, PRL, and calcitonin similarly have low LLPS propensity yet high amyloid aggregation propensity (*SI Appendix*, Fig. S14), in line with intuition from the literature that hormones can be functional amyloids (12).

Interestingly, several notable amyloid-forming proteins belong to a third class, with both regions of high LLPS *and* amyloid aggregation propensity. Tau, for example, has two regions with high amyloid aggregation propensity around residues 306 to 311 [called PHF6 (79)] and 275 to 280 (called PHF6*), and one region of moderate LLPS propensity around its proline rich-domain. Similarly, major prion protein (PrP) has three regions of exceptional amyloid aggregation propensity (around residue 180, N terminus, and C-terminus) and one region of exceptional LLPS propensity (around residue 90). Alpha synuclein also shows one region with high amyloid aggregation propensity around residue 70, and a region of moderate LLPS propensity at the C-terminus. Like the PrP prion, two functional yeast prions (86) called Sup35 and URE2 also fit the pattern of having regions with high LLPS propensity and high amyloid aggregation propensity (*SI Appendix*, Fig. S15). CPEB3, a prion-like RNA-binding protein involved in neuronal function and memory, also fits this pattern (*SI Appendix*, Fig. S16). Of note as well are TDP-43, FUS, TAF15, and HNRNPA1, neuronal RNA-binding proteins that also form amyloids in neurodegenerative diseases, and each of these proteins have prominent regions with extreme LLPS propensity (*SI Appendix*, Fig. S16). While most residues with such high LLPS propensity have an amyloid aggregation propensity near 0, some of these high LLPS residues in neuronal RNA-binding proteins have higher-than-expected amyloid propensities. These regions with higher-than-expected amyloid propensities may be influential in nucleating amyloid formation in a condensate (see Discussion).

It appears that our models do not typically score an IDR residue as having both a high LLPS propensity and a high amyloid propensity. However, some notable exceptions forming a fourth class of dual high propensity include Huntingtin’s N-terminal IDR (Fig. 4*vi*), which has a poly-Q stretch near the N terminus, and PMel17, which has a T-rich stretch. Huntingtin misfolds in Huntington’s disease and PMel17 is a functional amyloid that facilitates melanin synthesis. Glutamines are common in amyloid structures because their amide side chains form amide ladders that form H-bonds along the filament axis (87). Additionally, the multivalency of glutamine’s amide groups may promote the interactions necessary for LLPS. PolyQ-expansion in huntingtin is associated with Huntington’s disease and aberrant phase separation (88, 89), and we observe that both LLPS scores and amyloid aggregation residue scores increase when expanded from polyQ-21 to polyQ-50 (Fig. 4*vi*). Threonine can also form H-bonds along the filament axis of an amyloid, and they may also form multivalent interactions in a condensate as described by Rekhi et al. (43)

Finally, we apply the amyloid-predict model to single nucleotide polymorphisms (SNPs) in the IDRs of the human proteome and highlight several mutations that induce notable increases and decreases in amyloid aggregation propensities. Specifically, we compare aggregation scores of various fragments within 14aa of the mutation site to evaluate the effect of the mutation (see Methods). Pathogenic SNPs are enriched for larger predicted changes in aggregation propensity compared with benign variants (*SI Appendix*, Fig. S19). This enrichment is statistically significant (Fisher’s exact test across tail thresholds; odds ratios ~2 to 4 depending on threshold) and remains robust after controlling for potential confounding factors such as IDR length, net charge change, and hydrophobicity change (*SI Appendix*, Fig. S20). These findings suggest that disease-associated mutations are more likely to perturb aggregation-related biophysical features than benign polymorphisms. We did not perform an analogous SNP-level analysis for LLPS propensity changes in this study, due to its lower sensitivity to mutations.

Of the pathogenic SNPs that most increase aggregation propensities, several are related to disorders in muscle and cytoskeletal proteins (A649V in Myosin-7; P419S in Desmin; P209L in BAG3; S60F in Myotilin; T27I in LMNA), retinal and eye proteins (P870L in ABCA4; G614V in CRB1), or neurodegeneration (G54V in protein C19orf12; D300V in Erlin-2). Of the pathogenic SNPs that most decrease aggregation propensities, several are related to disorders in structural proteins (G1066V, G1066S, G1066D in collagen alpha-5(IV) chain; L12R in collagen alpha-1(I) chain; C3Y in CRIPT) and membrane/transport proteins (L15H in LDLR; I1922S in SCN1A). See *SI Appendix*, Fig. S21 for an example of how the G54V in C19orf12 causes large increases in aggregation propensity relative to WT.

## Discussion

amyloid-predict and LLPS-predict leverage ESM embeddings to score phase-transition propensity with remarkable speed and accuracy. Our amyloid models appear to match or exceed other amyloid predictors in accuracy on a hexapeptide benchmark (Fig. 1*D*), while being sensitive to single-point mutations and running significantly faster than a comparably performing model (*SI Appendix*, Table S1) in high throughput; our LLPS model, trained specifically on intrinsically disordered regions (IDRs), shows strong performance in identifying condensate-driving segments across the disordered human proteome. Because both models can score thousands of fragments in seconds on a single GPU, they enable proteome-scale annotation and rapid in-silico filtering of design candidates on a consumer GPU. Importantly, both amyloid-predict and LLPS-predict are freely available as open-source tools (github.com/samlobe/amyloid-predict and github.com/samlobe/LLPS-predict), enabling straightforward deployment and development in academic and commercial settings.

Due to their speed, these models integrate seamlessly into large-scale sequence design workflows, quickly flagging problematic or desirable motifs. Protein engineers can i) filter out fragments that threaten unwanted aggregation or phase separation, or ii) select for motifs that foster desirable behaviors—self-assembling nanomaterials, viscoelastic droplets, or phase-separating scaffolds. Kilgore *et al.* recently showed how a pLM-based classifier can guide protein design to target specific condensates in cells (69); our paired models extend that concept and enable guided design of proteins with specific LLPS or amyloid propensity. For example, sequence engineering guided by LLPS-predict may enable the design of synthetic membraneless organelles that can organize, sequester, or concentrate signaling molecules.

At the proteome level, the two scores illuminate how amyloidogenic and LLPS-driving motifs are distributed across functional and spatial categories, generating hypotheses for disease mechanisms and synthetic-biology targets. Fragments with high amyloid or LLPS scores can be further studied with experimental assays, molecular simulation, or structure prediction tools to better understand their functional role. Additionally, fragments with high LLPS scores, for example, can be artificially removed or appended to other proteins to investigate their role or alter their condensation properties.

Our findings suggest that even simple linear probes of mean-pooled pLM embeddings can reliably capture amyloidogenic and LLPS-related signals. The dataset used in training amyloid-prediction and LLPS-prediction models is certainly influential in the model’s behavior (*SI Appendix*, Fig. S1 and Tables S2 and S3), and the present model training strategy can easily be transferred to other datasets. Data quality remains a pivotal factor, and larger datasets are not inherently better: we demonstrate that using a broader subset of WALTZdb (i.e. including peptides that lack Th-T data) worsens performance (AUROC) on the same test set (*SI Appendix*, Fig. S1). As amyloid or LLPS datasets improve in both size and quality, they can be incorporated in future models.

LLPS-predict currently estimates LLPS propensity based on sequence features shared by known LLPS drivers, but it does not yet account for the influence of environmental factors such as pH or salt concentration. As richer datasets become available, future LLPS predictors could be trained to distinguish between different categories of phase separation—for example, homotypic versus heterotypic, UCST versus LCST, or LLPS triggered by specific ions or pH changes. Models that incorporate these context-dependent variables will provide researchers with more practical tools for probing and engineering IDRs with desired phase separation behaviors under defined conditions.

We chose to train our amyloid-predict and LLPS-predict models based on experimental data that reflect realistic in-vitro and in-vivo environments. Models can be trained with large, simulated datasets such as the spontaneous homotypic phase-separating IDRs reported in von Bülow, et al, (73) but the resulting models will have additional limitations due to a) force field accuracy (e.g., in modeling solvation effects or effective coarse-grained interactions), b) system size and timescale limitations, c) assumptions about the relevance of cofactors, and d) inaccuracies that compound as the pLMs approximate MD-based models versus experiment.

There are several avenues for improvement in future iterations at predicting amyloid aggregation and LLPS propensities of IDRs. For example, because pLM-based classifiers are inherently less interpretable than physics-based methods, we present a ten-feature hexapeptide amyloid classifier (called FETA) whose features may offer interpretable insights into biophysical drivers of amyloid aggregation: e.g., propensities to form cross-β hydrogen bonds, propensities to form sidechain zippers, water–protein interactions, docking and locking behavior of monomers, or other transitions in the free-energy landscape toward aggregation-prone states (90–93). Sparse autoencoders (94–96) may also be useful in providing interpretable insight into amyloid aggregation. In addition, instead of mean-pooling the embeddings of the final transformer layer, future models may utilize the information in the hidden layers and in each amino acid’s embeddings (e.g., via sliced-Wasserstein embeddings (97)). Although we observed no obvious performance trend with model size or type using the current datasets, larger and higher-quality amyloid/LLPS datasets could reveal nuanced advantages of specific architectures. Fine-tuning of pLMs may also improve models (98). Finally, posttranslational modifications—known to influence both amyloid formation and LLPS (99, 100)—could be incorporated by PTM-aware pLMs like PTM-Mamba (101). By refining these aspects—interpretability, PTM inclusion, data quality, and more comprehensive use of pLM embeddings—we envision an even more powerful and versatile framework for modeling protein phase transitions at scale, and these methods should nicely complement biophysical modeling and experimental characterization.

### IDRome Analysis.

When we apply our classifiers to the human IDRome, we observe interesting enrichment patterns for both aggregation propensity and LLPS propensity. For example, we observed that calcium-, heparin-, and carbohydrate-binding proteins, as well as certain signaling proteins such as GPCRs, frequently show elevated aggregation scores. One possible explanation is that self-association (e.g., via hydrogen bonding or hydrophobic IDR–IDR interactions) is adaptive for these protein families, and this behavior resembles that of the amyloids in the amyloid-predict training set. For heparin-binding proteins, the formation of vertical stacks of positively charged side chains (e.g., lysines) along an amyloid-like filament axis could be advantageous for binding polyanionic molecules like heparin; likewise, calcium-binding proteins may benefit from the amyloid-like stacking of negatively charged side chains to bind the positive, divalent calcium ions. In some extracellular or signaling contexts, transient clustering of proteins via IDRs is beneficial for function—for instance, signal amplification by oligomerized GPCRs (102–104). This clustering propensity may share biophysical aspects of amyloid propensity; however, some of the elevated aggregation propensities of GPCR IDRs may instead reflect hydrophobic stretches that facilitate membrane association.

In contrast, certain protein families showed elevated LLPS propensity, such as DNA- and RNA-associated protein families and nuclear proteins generally (e.g., nuclear matrix, nuclear speck, and chromatin). Structural molecule activity proteins also had elevated LLPS propensity. Many of these IDRs have large, low-complexity domains and multivalent interaction motifs that can facilitate liquid droplet formation (105). These observed LLPS enrichments are in line with an expanding body of experimental data. For instance, many of the top-scoring DNA- and RNA-associated proteins—including FUS, TAF15, TDP-43, and hnRNPA (*SI Appendix*, Fig. S16)—are known stress-granule scaffolds whose phase separation has been confirmed by in vitro droplet assays and live-cell imaging (106–108). Likewise, transcriptional co-activators such as MED1 and RNA polymerase II, which were ranked highly by LLPS-predict (Fig. 3 *C*, *i* and *SI Appendix*, Fig. S15), have been shown to form liquid-like condensates that compartmentalize transcriptional “hubs” (109–111). Recent polymer-physics modeling further delineates domain-specific sequence determinants underlying MED1’s condensation behavior (112). Even structural proteins—ranging from the nuclear-matrix scaffold SAF-A to cytoskeletal organizers such as the microtubule-binding protein tau and the actin-nucleating DIAPH3—were rated highly by our LLPS classifier (Fig. 3 and *SI Appendix*, Fig. S17) and have been seen to assemble into viscoelastic condensates (113–116), perhaps to better recruit their condensate partners or to buffer mechanical stress. Our purely sequence-based predictor successfully identifies these experimentally validated condensate drivers, despite their diverse interactions and functions, thus supporting the view that multivalent, low-complexity motifs encode a conserved “molecular grammar” for droplet formation.

A small subset of proteins has IDRs with high scores for both LLPS and amyloid aggregation metrics, and this set includes famous prionic proteins. Mammalian PrP, yeast prions SUP-35 and URE2 (*SI Appendix*, Fig. S15), and neuronal tau have been identified as prion-like and have been observed to form both liquid-like droplets and amyloid fibrils. In some cases, liquid-droplets appear to form reversibly (117, 118), but in the case of specific mutations, or upon a change in concentration, pH, phosphorylation, or another stressor, these liquid-like droplets can “harden” into β-sheet rich fibrils; an experimentally observed “condensate-to-amyloid” route (119–121). As such, both liquid-like condensates and amyloid fibrils can therefore represent alternative minima on a shared energy landscape (92, 122, 123). For example, as identified by LLPS-predict and shown experimentally, tau’s proline-rich domain appears to drive LLPS [see Zhang, et al. (81)], possibly playing a physiological role by concentrating tau proteins at the microtubule surface and thereby facilitating their binding. Conversely, as identified by amyloid-predict and shown experimentally, the microtubule-binding domain (driven by PHF6 and PHF6*) promotes fibril formation. Tau hence appears to exist in a delicate balance (a state of “frustration”) between a potentially physiological LLPS tendency and a pathological amyloid tendency, with mutations or changes in the environment capable of shifting the balance from liquid-droplet to solid-fibril.

We expect future research to investigate mechanisms of prion formation and how the balance of amyloid propensity and LLPS propensity affects a protein’s prionic properties. Considering the pattern of prion proteins containing separate amyloid-prone and LLPS-prone segments, it would be insightful to miniaturize a protein by connecting its two minimal elements necessary for controlled prionic activity to comprehensively investigate its phase behavior. Such a miniaturized prionic protein could enable studies to precisely determine the environments (e.g., ions, cofactors, pH, and crowding) that initiate droplet formation and the transition to amyloid formation. Studies may alter the LLPS-prone segment to have higher or lower LLPS propensity (according to LLPS-predict) to better understand if LLPS protects from amyloid formation or facilitates it.

While we have highlighted prionic proteins with separate segments that appear responsible for LLPS and amyloid formation, there are a few examples of segments in the IDRome that score highly for LLPS and amyloid propensity at the same time. The poly-Q tract in the N terminus of the Huntingtin protein (Fig. 4*vi*), the T-rich regions of functional amyloid Pmel17 (*SI Appendix*, Fig. S16*F*), and S-rich regions of MED1 (*SI Appendix*, Fig. S17) score highly for both LLPS and amyloid propensity. The poly-Q tract has been seen to form reversible condensates that nucleate pathological fibrils as poly-Q length or crowding increases (89, 124). Similarly, Pmel17’s RPT domain with many threonines has been seen to form liquid condensates that are responsive to salt concentration and pH before transitioning to solid aggregates (125). Glutamine’s bifunctional amide side chain and the dense network of hydroxyl H-bonds in threonine- and serine-rich stretches supply the multivalent contacts that drive LLPS, yet the same side-chain hydrogen bonds can line up along the β-sheet axis to lock these segments into amyloid fibrils. Our model’s continuous scoring captures the “spectrum” of phase transition propensities, and this framework emphasizes that there is a continuum between purely liquid and solid states that is sensitive to sequence and environment. It may be useful to pinpoint regions that drive these two phenomena, as they can be targeted to steer equilibrium away from phases that are linked to disease.

amyloid-predict and LLPS-predict can also be used to develop testable hypotheses in biomedical research. For example, amyloid-predict can be used to prioritize amyloidogenic IDRs and LLPS-predict can be used to prioritize LLPS-driving IDRs therapeutically, perhaps using recently developed IDR-targeting protein design tools (126–128). It may be useful to target disordered amyloidogenic IDRs rather than a specific amyloid interface; amyloid structures are often polymorphic and heterogeneous (129) so inhibiting one amyloid interface may simply favor other equally toxic interfaces while targeting the disordered IDR may prevent toxic interfaces from forming.

As a case study, we apply amyloid-predict to proteins linked to Alzheimer’s disease and uncover IDR fragments that can be prioritized for mechanistic wet lab studies. Specifically, we calculate the aggregation scores for the IDRs in TMEM132C and complement receptor 2 (CR2)—two of the proteins with the strongest positive associations to Alzheimer’s disease in a recent GWAS meta-analysis (130)—and find highly amyloidogenic windows around TMEM132C residues 1,060 to 1,080 and CR2 residues 980 to 995 (*SI Appendix*, Fig. S18). These short stretches could, in principle, nucleate pathological phase separation cascades that facilitate Aβ and tau aggregation, the hallmarks of Alzheimer’s disease. We highlight them here as testable targets that can be targeted with IDR-binders (126–128) and investigated with peptide seeding experiments or in-cell aggregation assays, illustrating how sequence-level scores can rapidly generate actionable hypotheses for neurodegenerative disease research.

## Conclusion

The present work introduces a unified, sequence-based framework for quantifying two of the most consequential phase-transition behaviors in proteins—amyloid aggregation and LLPS—at a speed that enables proteome-level analysis in hours on a single GPU. We couple fast protein language modeling with lightweight logistic-regression classifiers, called amyloid-predict and LLPS-predict. We demonstrate that ESM’s pLMs can i) match or exceed the accuracy of physics-based tools, ii) run orders of magnitude faster, and iii) remain sensitive to subtle mutations for the amyloid models.

Applying these models to the ~28 k IDRs in the human IDRome reveals enriched amyloid aggregation or LLPS propensity in specific molecular function and cellular localization categories—most notably in GPCRs, carbohydrate-binding, RNA-, and DNA-binding proteins. A small but illuminating subset of proteins have IDRs that score highly on both scales, including some famous prionic proteins.

These models may enable rapid prioritization of novel proteins with interesting phase separation properties and programmable tuning of amyloid or LLPS motifs for synthetic biology or materials applications. Looking ahead, richer training sets and more exploitation of residue embeddings and hidden layers of pLMs should further sharpen predictive power. We anticipate that integrating these fast data-driven models with physics-based simulation and complementary experimental approaches will accelerate the rational design and control of functional and pathological protein phase transitions.

## Methods

### Datasets.

Amyloid classifiers were trained on several peptide sets: 41 tau fragments (15aa) with ThT/pFTAA assays, 70 peptides (10aa) from PrP/lysozyme/β2-microglobulin, 222 WALTZdb hexapeptides (6aa), a reduced 10-feature model (FETA) from the 6aa set, a combined 6/10/15aa model, and 20aa peptides from Thompson *et al.* 15aa peptides were labeled positive if either ThT or pFTAA fluorescence exceeded the control by 50%; other datasets’ labels were from the original sources. For LLPS, we used 28,058 intrinsically disordered regions (IDRs) from the human proteome [Tesei et al. (75)], with positives defined as 180 IDRs from 118 CD-CODE LLPS drivers. See *SI Appendix*, Appendix S1.

### Model Training.

Sequences were embedded with ESM2-3B (2560-dimensional per residue). Mean-pooling across residues yielded a single 2560-vector per peptide or IDR, standardized before model fitting. Because ESM embeddings are contextualized by the full sequence, this pooled vector implicitly encodes sequence order and patterning even though positional information is averaged. Other pLMs’ embeddings were used for benchmarking such as ESM2-650 M and -15B; ESMC-300 M, -600 M, -6B; and esm3-small, -medium, -medium-multimer, and -large. We used logistic regression with L2 regularization. For the combined amyloid model, class and dataset weights were balanced, with nested cross-validation (outer LOOCV; inner split to select C). The 15aa and 10aa sets were trained directly with C = 1 due to limited positives. For the 6aa set, feature selection was performed with L1, leading to two models: a 24-feature “6aa best” and a 10-feature FETA. L2 strength was tuned by cross-validation. LLPS classifier training used stratified validation (20%) and two-stage regularization (L1 for feature selection, L2 for final model), with performance estimated over 30 random splits. Performance was reported as ROC-AUC and average precision. Comparisons to existing amyloid predictors (WALTZ, TANGO, PASTA, CANYA) were made on the 6aa dataset. Error bars reflect 16th–84th percentile values from repeated train/test splits. Baseline methods were evaluated using their continuous scores and ROC-AUC (all thresholds), rather than a single threshold; see *SI Appendix*, Appendix S1 for the bootstrap procedure and score-handling details.

### Per-Residue Scoring.

Residues were scored by tiling IDRs into overlapping fragments (amyloid: 6/10/15aa; LLPS: 15/25/40aa) and averaging scores of all fragments covering that residue. Scores across probe lengths were averaged for the final per-residue profile. See *SI Appendix*, Appendix S2.

### Proteome-Scale Annotation (IDRome).

We applied the per-residue workflow to all ~28 k human IDRs (Tesei *et al.* definition). For category analyses, residues with score >0.5 were counted as high-propensity. We summarized by Gene Ontology molecular function and cellular component categories, reporting the fraction of high-propensity residues in each category. To probe composition vs. patterning, we computed composition-averaged scores (amino acid frequencies × per-residue mean scores) and compared with observed fractions.

### SNP Analysis.

For each single-nucleotide polymorphism (SNP) within an IDR, we computed amyloid per-residue scores for the wild-type and the mutant sequence and compared local windows spanning ±14 residues around the variant. Differences between pathogenic and benign SNPs were assessed using Fisher’s exact tests on tail enrichment of |Δaggregation| values, with odds ratios and bootstrap CI reported. Δaggregation values were computed by measuring the difference in the average per-residue scores of the IDR (i.e. IDR residues within 14aa of the SNP site) with and without the SNP. Additional analyses controlled for potential biases including IDR length, net charge change, and hydrophobicity change. A representative high-shift example is shown in *SI Appendix*, Fig. S21.

### Implementation.

ESM2 embeddings were computed on a single RTX3090 GPU (24 GB VRAM), except for the ESM2-15B model’s embeddings which were computed on a V100 GPU. ESM3 and ESMC embeddings were computed on EvolutionaryScale’s cluster. Classifiers used scikit-learn. Scripts and pretrained models are available on GitHub (github.com/samlobe/amyloid-predict and github.com/samlobe/LLPS-predict) under permissive licenses for academic and commercial use.

## Supplementary Material

Appendix 01 (PDF)

## Data Availability

Pretrained models, scripts, and datasets have been deposited in Zenodo as archival snapshots with DOIs (131, 132). The corresponding live GitHub repositories are publicly accessible at https://github.com/samlobe/amyloid-predict and https://github.com/samlobe/LLPS-predict. All other data are included in the article and/or *SI Appendix*.
