# Supplementary material for: amyloid-predict and LLPS-predict: Predicting phase separation propensities in the intrinsically disordered proteome
Source: Proc Natl Acad Sci U S A. 2026 May 26;123(22):e2531932123. doi: 10.1073/pnas.2531932123 (PMC13229271; doi:10.1073/pnas.2531932123)
Supplement: Supplementary file 1 — Appendix 01 (PDF) [file pnas.2531932123.sapp.pdf]

PNAS

Supporting Information for

amyloid-predict and LLPS-predict: Predicting Phase Separation Propensities in the Intrinsically Disordered Proteome

Samuel Lobo, Leif Griem, M. Scott Shell, and Joan-Emma Shea

Corresponding Authors:
M. Scott Shell
Email: shell@ucsb.edu

Joan-Emma Shea
Email: shea@chem.ucsb.edu

This PDF file includes:

Appendix S1: protein language model classifier training
Appendix S2: per-residue scoring
Figures S1 to S21
SI References

Appendix S1: protein language model classifier training

With **amyloid-predict** we share six logistic regression amyloid classification models trained with ESM2-3B embeddings:

- a **15aa model** trained on 41 tau fragments from Louros, et. al. (1);
- a **10aa model** trained on 70 fragments (2) from PrP, lysozyme, and β_2 microglobulin;
- a **6aa model** trained on 222 peptides from WALTZdb with Th-T data;
- another 6aa model with just 10-feature called **FETA** (Fast ESM-based Ten-feature Amyloid classifier);
- a **general** model trained on the 6aa, 10aa, and 15aa datasets; and finally,
- a **20aa model** trained on random 20aa peptides from Thompson, et. al. (3)

For the 15aa dataset, we considered a peptide to be in the positive class if the mean signal from either the Th-T data or the pFTAA data was 50% greater than the signal from the control. The labels for the 6aa, 10aa, and 20aa were taken from the original publications.

Additionally, with **LLPS-predict** we share a logistic regression *LLPS driver* classification model for IDRs, trained with ESM2-3B embeddings (4). Its dataset was 28,058 IDRs, identified by Tesei, et. al. (5) using window-averaged pLDDT confidence scores from AlphaFold predictions of the human proteome to identify IDRs. The IDRs had lengths ranging from 30 to 1496 with a median of 80 amino acids. We construct the positive IDR class by first identifying 118 LLPS driver proteins that were identified from the CD-CODE database (6) and selecting their 180 IDRs as enriched in LLPS propensity. The negative IDR class consisted of the remaining 27,878 IDRs from the proteome.

For each model we extract embeddings from the peptide using ESM protein language models, specifically 2560 embeddings per residue with ESM2-3B for the bulk of our analysis and the models shared on GitHub. We mean-pool these embeddings, i.e. averaging the 2560 x L embeddings matrix over L amino acids to get a single 2560 x 1 embedding vector for each sequence. We then standardize each embedding (i.e. zero mean and unit variance) and train logistic regression models as described below. Other ESM3 and ESMC models (Fig S2-4, S7) used in benchmarking have varying numbers of embeddings (7).

<u>**general** amyloid model training</u>

We balance the weights of each of the three datasets (6aa, 10aa, 15aa), and we balance the weights of each class (amyloid vs non-amyloid) in each dataset. Feature dimensions were standardized within each fold. Model selection uses a nested cross-validation scheme: in the *outer* loop leave-one-out cross validation (LOOCV) held out a single peptide for testing while the *inner* loop was used to select the L2 regularization strength. The inner loop features a 2-fold stratified split to select the inverse regularization parameter *C* from {1, 0.1, 0.01} that maximized the average precision. The model fitted with the optimal *C* was then evaluated on the corresponding outer test fragment, yielding out-of-sample probability estimates for every peptide and a distribution of best-*C* values. After cross-validation, we retrain a final model on the full dataset using the modal *C* (0.1). This nested cross-validation scheme was done to guard against data leakage so that hyperparameter selection and model performance better reflect the classifier's ability to generalize to unseen peptide sequences.

<u>**15aa** and **10aa** amyloid model training</u>

The 15aa and 10aa models were trained identically, without feature selection or hyperparameter tuning considering that their datasets had only ten and eleven positive samples, respectively. A standard inverse regularization parameter of *C* = 1 was used, and model performance was estimated using LOOCV.

<u>6aa amyloid model training</u>: **FETA** model and best **6aa** model

Considering the larger sample size of 222 peptides, we chose to do feature selection and tune L2 regularization strength. A validation set of 30% was used. We first measure ROC AUC and Average Precision for a range of L1 regularization strengths and observed that performance peaked around 24 features, and that ~10 features had a high performance considering its small feature set. Therefore, we trained two models: one called **6aa best** with 24 features and another called **FETA** (Fast ESM-based Ten-feature Amyloid classifier) with 10 features. The ten features were embeddings 260, 278, 897, 1079, 1162, 1353, 1522, 1806, 1917, and 2119 (0-indexed) from the ESM2-3B model. 5-fold cross validation was used to tune the L2 regularization parameter *C* from {0.01, 0.05, 0.1, 0.5, 1, 5} such that the mean F1 score between folds was maximized. The final models were trained using the selected L2 regularization strength with the previously selected feature set on the full dataset.

<u>Comparison to other amyloid models</u> (Fig 1D)

We compare our 6aa protein language model (pLM) amyloid classifier to other physics-based and statistical amyloid classifiers: WALTZ, TANGO, PASTA parallel, PASTA antiparallel, and CANYA. To do this we used the 222 6aa peptide dataset and trained one-feature logistic regression models using the set of scores from each of those algorithms, and we compare ROC AUC for each model. Because ROC AUC depends only on rank ordering, results are unchanged whether we evaluate ROC AUC on the baseline scores directly versus the logistic regression recalibration (mapping the baseline scores onto a probability scale). To evaluate our confidence on these predictions, we repeated 500 random train-test splits and computed ROC on the heldout split; we show the median, 16th percentile, and 84th percentile ROCs in Fig 1D. We compare the ROC AUC without feature selection (i.e. using all 2560 features) versus with the 24 features in the **6aa best** model, and we demonstrate that performance with the pLM approach exceeds the other models while requiring substantially less computation: in benchmarks ~10-60x faster than PASTA web-server wall-clock for matched conditions (Table S1).

Length (aa)	N seq	amyloid-predict batches (sizes)	amyloid-predict pred time (s)	amyloid-predict throughput (seq/s)	PASTA server wall time (s)	PASTA throughput (seq/s)
6	1	1 (1)	1.584	0.63	16	0.06
6	10	1 (10)	1.594	6.27	28	0.36
6	50	1 (50)	1.649	30.32	39	1.28
6	100	1 (100)	1.771	56.47	40	2.50
6	500	1 (500)	2.583	193.57	163	3.07
25	1	1 (1)	1.528	0.65	27	0.04
25	10	1 (10)	1.615	6.19	16	0.62
25	50	1 (50)	1.496	33.42	36	1.39
25	100	1 (100)	1.844	54.23	53	1.89
25	500	4 (157,157,157,29)	5.025	99.50	160	3.12

Table S1. Throughput benchmark comparing amyloid-predict (GPU) to PASTA web server wall time. Amyloid-predict and LLPS-predict are essentially the same speed, controlling for sequence length. Amyloid-predict was run on a NVIDIA RTX 3090 Ti GPU (toks-per-batch setting fixed at 4096, which sets the batch sizes). Reported amyloid-predict times correspond to the tool's "Prediction time (s)" and exclude one-time model initialization; the mean weight-loading time was approximately 10.5 s on GPU. For reference, on a Mac CPU (M1 chip) the mean weight-loading time was approximately 64 s, and GPU prediction was ≈40× faster than Mac CPU prediction (median speedup across matched conditions; range ≈20×–80×). PASTA timings are stopwatch wall-clock measurements from the public web server (includes network latency and model initialization). Amyloid-predict and LLPS-predict are available on GitHub (github.com/samlobe/amyloid-predict and github.com/samlobe/LLPS-predict) and open-sourced, unlike PASTA.

Cross-dataset transfer evaluation

Held-out dataset (test)	Train datasets	Held-out dataset (test)	ROC-AUC
6aa	10aa + 15aa	6aa	0.741
10aa	6aa + 15aa	10aa	0.703
15aa	6aa + 10aa	15aa	0.716

Table S2. Leave-one-dataset-out (LODO) evaluation of the general amyloid-predict model. Model trained on two datasets (evenly weighted) and evaluated on the held-out third dataset. Performance is reported as ROC-AUC.

Train model	Test: 6aa dataset	Test: 10aa dataset	Test: 15aa dataset
6aa-best model		0.613	0.784
10aa model	0.616		0.494
15aa model	0.640	0.653	

Table S3. Cross-dataset transfer evaluation of single-length amyloid-predict models. Each model is trained on a single dataset (6aa, 10aa, 15aa) and evaluated on the other datasets. Performance is reported as ROC-AUC.

LLPS driver IDR classifier training:
A stratified validation set of 20% was used, containing equal proportions of the positive and negative classes. A grid search was done to select a set of features and pick a L2 regularization strength: C_1 values from {0.01, 0.05, 0.1, 0.5} were used for feature selection, and C_2 values from {1, 0.1, 0.01, 0.001, 0.0001} were used for regularizing the selected features. For each combination of C_1 and C_2 values, ROC-AUC was evaluated in each fold (5-fold cross validation within the training set), and the combination of C_1 and C_2 values that had the largest mean ROC AUC was set aside. The weights were balanced for each class to improve model calibration, considering that many LLPS driver IDRs in the proteome have not yet been discovered. The selected C_1 and C_2 parameters were then used for feature selecting and regularization on the full 80% training set and evaluated on the 20% validation set to measure model performance. Finally, the same C_1 and C_2 were used on the full dataset to make the final model. To fairly evaluate model performance, we used thirty random training-validation splits and reported the median ROC AUC along with the 16th an 84th percentile ROC (error bars) on the validation sets (Fig 1F).

Robustness to functional-category bias (leave-one-molecular-function-out)

Because LLPS-predict's positive class is derived from curated LLPS driver proteins (CD-CODE), performance could in principle be inflated by over-represented, well-studied functional classes (e.g., RNA/nucleic-acid binding proteins) rather than reflecting broadly transferable sequence signals. To probe this, we performed a leave-one-GO-molecular-function-out evaluation: for each well-represented GO molecular function term (≥20 IDRs in the positive class), we removed all IDRs annotated with that term from the training set, repeated the same model-selection procedure on the remaining data, then evaluated on the held-out term.

Across 19 eligible molecular function terms, held-out performance remains substantial (median test ROC-AUC 0.73, range 0.61–0.85). Notably, "nucleic acid binding" comprises 103/180 positive IDRs and 6509 negatives; when this entire category is withheld from training, LLPS-predict still achieves a held-out ROC-AUC of 0.73, indicating that performance is not driven solely by a single dominant functional class. This analysis supports the claim that LLPS-predict captures sequence signals that transfer across diverse functional annotations. Because each IDR can have multiple GO annotations, we treat these as a set of robustness tests (one per term) rather than pooling them into a single cross-validated estimate.

heldout molecular function GO category	n_test_pos	n_test_neg	test AUC
nucleic acid binding	103	6509	0.730
enzyme binding	73	3486	0.677
identical protein binding	71	2889	0.697
rna binding	67	2440	0.763
dna binding	65	4441	0.731
molecular adaptor activity	59	779	0.786
protein-containing complex binding	51	2956	0.797
mrna binding	38	485	0.689
chromatin binding	38	1137	0.694
metal ion binding	33	6767	0.771
transcription coregulator activity	31	867	0.641
sequence-specific dna binding	31	3004	0.845
protein domain specific binding	30	1168	0.647
protein kinase binding	29	1190	0.704
signaling receptor binding	28	2043	0.789
transcription coactivator activity	24	488	0.620
sequence-specific double-stranded dna binding	24	2782	0.840
dna-binding transcription factor binding	22	826	0.607
transcription cis-regulatory region binding	22	2674	0.818

Table S4. Leave-one-GO-molecular-function-out evaluation for LLPS-predict. For each GO molecular function term meeting inclusion criteria (>20 IDRs in positive class), we trained LLPS-predict on the remaining IDRome IDRs and evaluated on the held-out term. Table reports held-out test sizes and ROC-AUC.

Composition analysis

Composition-preserving scrambling analyses were performed by permuting residues within selected sequences while maintaining identical amino-acid composition. For permutation studies of short amyloidogenic segments (e.g., tau PHF6), all possible permutations were evaluated. For longer sequences, multiple random scrambles were generated. Predictions were compared between native and scrambled sequences using both probability scores and logistic regression logits.

Appendix S2: per-residue scoring

For per-residue aggregation scores using amyloid-predict, we break down each IDR into overlapping fragments of length 6aa, 10aa, and 15aa, due to the model's experience with predicting amyloids of these sizes in its training data. For per-residue LLPS scores using LLPS-predict, we break down each IDR into overlapping fragments of length 15aa, 25aa, and 40aa; the 15aa fragments enables sensitivity to subtle mutation effects, while the 25aa and 40aa fragments better identify long-ranged patterns typical of LLPS. See Fig S10 to view how this fragment probe lengths affect the scores for tau and TDP-43. Users may choose to adjust probe lengths; probe lengths have varying sensitivities to local and long-range interactions; shorter length probes for LLPS-predict may be less suspectable to model memorization yet may lack important long-range context.

For a given fragment probe length, a residue is given a score by averaging the scores of each fragment that the residue is in; see Fig 2A. For example, residue 3's score would be the average of the fragments scores for fragment 1-15, 2-16, and 3-17. Finally, the per-residue scores for each of the three fragment probe lengths are averaged together to get a final per-residue score. See amyloid-predict-per-res and llps-predict-per-res commands in the GitHub repos for a python implementation of the above description.

For our IDRome analysis, the 6aa fragments had a "sliding window" of 1 (e.g. 1-6, 2-7, etc.), the 10aa fragments had a sliding window of 2 (e.g. 1-10, 3-12, etc.), the 15aa fragments had a sliding window of 3, the 25aa fragments had a sliding window of 4, and the 40aa fragments had a sliding window of 5.

For GO analyses, we use a fixed threshold (0.5) for defining frac_high (i.e. fraction of residues with high amyloid or LLPS propensity); robustness to threshold choice (0.4–0.6) and IDR-level confounder controls (hydrophobicity and IDR length) are summarized in Tables S5–S6.

While Figure 3 shows a *residue*-level analysis of model predictions for GO categories, while Table S5 shows a complementary *IDR*-level analysis to avoid deflating p-values. Specifically, for each GO category we compared the distribution of per-IDR frac_high values against all remaining IDRs using the Brunner–Munzel test with Benjamini–Hochberg false discovery rate (FDR) correction. Under this IDR-weighted analysis, the Golgi lumen category does not reach statistical significance for LLPS propensity, indicating that its elevated residue-level signal is driven primarily by a subset of longer IDRs; nevertheless, it remains of interest because many residues in this category exhibit high predicted LLPS propensity.

To assess whether GO enrichments could be explained by simple compositional effects, we performed IDR-level linear regression analyses controlling for mean Kyte–Doolittle hydrophobicity and IDR length (Table S6). The outcome variable was frac_high, and predictors included GO category membership together with mean Kyte–Doolittle hydrophobicity and IDR length. Regression coefficients for GO membership therefore represent associations with aggregation or LLPS propensity after accounting for these potential confounders. Statistical significance was assessed using standard linear-model p-values with Benjamini–Hochberg FDR correction across all tested GO category/propensity combinations.

The Brunner–Munzel test here evaluates whether IDRs in a GO category tend to have larger frac_high values than the background IDRome without assuming equal variances. The associated rank-biserial correlation (RBC) provides an effect-size estimate for this enrichment, where positive values indicate that IDRs in the GO category are more likely than background IDRs to have higher frac_high values (and negative values the opposite). FDR-adjusted q-values account for multiple GO-category comparisons. Regression coefficients in Table S6 should be interpreted separately from RBC effect sizes, as they reflect associations after covariate adjustment rather than direct enrichment magnitudes.

GO category	phase propensity type	GO term	# IDRs	effect size (RBC @0.5)	FDR q-value (@0.5)	effect size range (0.4–0.6)
molecular_function	amyloid	G protein-coupled receptor activity	745	0.207	8.38e-17	0.191–0.213
molecular_function	amyloid	transmembrane signaling receptor activity	1562	0.288	8.24e-67	0.276–0.290
molecular_function	amyloid	signaling receptor activity	1902	0.267	7.51e-70	0.255–0.271
molecular_function	amyloid	carbohydrate binding	317	0.249	1.45e-11	0.236–0.255
molecular_function	amyloid	growth factor activity	204	0.196	1.88e-05	0.179–0.198
molecular_function	amyloid	calcium ion binding	1272	0.228	6.22e-40	0.213–0.230
molecular_function	amyloid	heparin binding	292	0.263	8.81e-12	0.251–0.263
molecular_function	amyloid	metalloendopeptidase activity	216	0.175	5.81e-05	0.175–0.197
molecular_function	amyloid	protein tyrosine kinase activity	349	0.197	1.64e-08	0.192–0.201
molecular_function	LLPS	mRNA binding	523	0.381	1.52e-45	0.294–0.490
molecular_function	LLPS	transcription coactivator activity	512	0.254	7.74e-24	0.201–0.310
molecular_function	LLPS	RNA binding	2507	0.203	2.14e-75	0.150–0.278
molecular_function	LLPS	transcription coregulator activity	898	0.233	1.08e-35	0.162–0.301
molecular_function	LLPS	chromatin binding	1175	0.209	1.44e-39	0.130–0.284
molecular_function	LLPS	SH3 domain binding	225	0.19	1.02e-07	0.094–0.231
cellular_component	amyloid	external side of plasma membrane	497	0.306	5.70e-26	0.306–0.310
cellular_component	amyloid	receptor complex	850	0.282	6.77e-37	0.264–0.287
cellular_component	amyloid	cell surface	1378	0.263	2.45e-51	0.262–0.263
cellular_component	amyloid	Golgi lumen	213	0.287	2.77e-11	0.281–0.288
cellular_component	amyloid	lysosomal membrane	545	0.064	1.89e-02	0.040–0.081
cellular_component	amyloid	Golgi membrane	862	0.133	5.98e-10	0.120–0.133
cellular_component	amyloid	late endosome	425	0.063	4.56e-02	0.043–0.080
cellular_component	amyloid	lysosome	919	0.080	1.71e-04	0.060–0.098
cellular_component	LLPS	ribonucleoprotein complex	876	0.207	1.16e-27	0.170–0.271

GO category	phase propensity type	GO term	# IDRs	effect size (RBC @0.5)	FDR q-value (@0.5)	effect size range (0.4–0.6)
cellular_component	LLPS	nuclear matrix	263	0.247	3.79e-12	0.156–0.329
cellular_component	LLPS	nuclear speck	715	0.260	7.70e-35	0.170–0.324
cellular_component	LLPS	transcription regulator complex	878	0.238	4.90e-37	0.144–0.327
cellular_component	LLPS	nuclear body	1456	0.200	3.44e-45	0.123–0.267
cellular_component	LLPS	chromatin	2439	0.215	3.07e-85	0.125–0.294
cellular_component	LLPS	nucleoplasm	6595	0.162	4.09e-129	0.095–0.237
cellular_component	LLPS	Golgi lumen	213	-0.029	3.38e-01	-0.045–-0.028
cellular_component	LLPS	kinetochore	263	0.095	2.36e-03	0.055–0.207
cellular_component	LLPS	bicellular tight junction	247	0.108	9.07e-04	0.065–0.160

Table S5. Robustness of GO enrichment results across aggregation/LLPS score thresholds. Gene Ontology (GO) categories highlighted in Fig. 3 are shown with effect sizes and statistical significance from the IDR-level enrichment analysis. Effect size is reported as the rank-biserial correlation (RBC) from the Brunner–Munzel test comparing the fraction of residues exceeding the per-residue score threshold within IDRs belonging to each GO category versus all other IDRs. P-values were corrected for multiple comparisons using the Benjamini–Hochberg false discovery rate (FDR) procedure across all tested GO terms at the 0.5 threshold. To assess sensitivity to the arbitrary cutoff used in Fig. 3, the range of effect sizes observed when varying the threshold from 0.4–0.6 is also reported. The number of IDRs annotated to each GO term is shown (# IDRs). Results are qualitatively stable across thresholds, supporting the robustness of the highlighted enrichments. Unlike Fig. 3, which summarizes residue-weighted fractions, the statistical analysis here weights IDRs equally.

GO category	phase propensity type	GO term	# IDRs	raw effect size (RBC)	raw q-value	adjusted β_GO	adjusted q_GO
molecular function	amyloid	transmembrane signaling receptor activity	1562	0.288	8.24e-67	0.0443	1.52e-34
molecular function	amyloid	signaling receptor activity	1902	0.267	7.51e-70	0.0403	4.73e-36
molecular function	amyloid	heparin binding	292	0.263	8.81e-12	0.0479	1.23e-09
molecular function	amyloid	carbohydrate binding	317	0.249	1.45e-11	0.0313	2.25e-06
molecular function	amyloid	calcium ion binding	1272	0.228	6.22e-40	0.0271	3.58e-15
molecular function	amyloid	G protein-coupled receptor activity	745	0.207	8.38e-17	0.0414	3.80e-13
molecular function	amyloid	protein tyrosine kinase activity	349	0.197	1.64e-08	0.0284	1.06e-05
molecular function	amyloid	growth factor activity	204	0.196	1.88e-05	0.0231	6.78e-03

GO category	phase propensity type	GO term	# IDRs	raw effect size (RBC)	raw q-value	adjusted β_GO	adjusted q_GO
molecular function	amyloid	metalloendopeptidase activity	216	0.175	5.81e-05	0.0170	5.39e-02
molecular function	LLPS	mRNA binding	523	0.381	1.52e-45	0.1936	1.02e-37
molecular function	LLPS	transcription coactivator activity	512	0.254	7.74e-24	0.0981	1.64e-16
molecular function	LLPS	transcription coregulator activity	898	0.233	1.08e-35	0.0803	6.78e-21
molecular function	LLPS	chromatin binding	1175	0.209	1.44e-39	0.0608	2.66e-18
molecular function	LLPS	RNA binding	2507	0.203	2.14e-75	0.0851	8.40e-56
molecular function	LLPS	SH3 domain binding	225	0.190	1.02e-07	0.0395	5.67e-03
cellular component	amyloid	external side of plasma membrane	497	0.306	5.70e-26	0.0498	1.25e-17
cellular component	amyloid	Golgi lumen	213	0.287	2.77e-11	0.0333	2.28e-04
cellular component	amyloid	receptor complex	850	0.282	6.77e-37	0.0386	8.70e-19
cellular component	amyloid	cell surface	1378	0.263	2.45e-51	0.0376	5.12e-27
cellular component	amyloid	Golgi membrane	862	0.133	5.98e-10	0.0153	5.86e-05
cellular component	amyloid	lysosome	919	0.080	1.71e-04	0.0066	9.46e-02
cellular component	amyloid	lysosomal membrane	545	0.064	1.89e-02	-0.0011	8.25e-01
cellular component	amyloid	late endosome	425	0.063	4.56e-02	0.0061	2.83e-01
cellular component	LLPS	nuclear speck	715	0.260	7.70e-35	0.0797	4.39e-17
cellular component	LLPS	nuclear matrix	263	0.247	3.79e-12	0.0980	7.71e-09
cellular component	LLPS	transcription regulator complex	878	0.238	4.90e-37	0.0720	7.34e-20
cellular component	LLPS	chromatin	2439	0.215	3.07e-85	0.0539	2.09e-34
cellular component	LLPS	ribonucleoprotein complex	876	0.207	1.16e-27	0.1105	1.78e-26
cellular component	LLPS	nuclear body	1456	0.200	3.44e-45	0.0572	1.31e-19
cellular component	LLPS	nucleoplasm	6595	0.162	4.09e-129	0.0473	3.17e-63
cellular component	LLPS	bicellular tight junction	247	0.108	9.07e-04	0.0209	5.39e-02

GO category	phase propensity type	GO term	# IDRs	raw effect size (RBC)	raw q-value	adjusted β_GO	adjusted q_GO
cellular component	LLPS	kinetochore	263	0.095	2.36e-03	0.0389	9.47e-03

Table S6. For GO categories highlighted in Fig. 3, IDR-level linear regression analyses were performed to assess whether aggregation and LLPS enrichments could be explained by simple compositional factors. The outcome variable was the fraction of residues in each IDR exceeding the scoring threshold (frac_high; threshold = 0.5). Models included GO category membership together with mean Kyte–Doolittle hydrophobicity and IDR length as predictors. Raw effect sizes (rank-biserial correlation; RBC) and FDR-adjusted q-values correspond to the enrichment analysis reported in Table S5, whereas adjusted regression coefficients (β_GO) and q-values reflect associations after controlling for hydrophobicity and IDR length. Note that the β coefficient for the GO term is directly related to the frac_high and should not be compared directly to the RBC. Because hydrophobicity is a well-established driver of aggregation and is partially encoded in amyloid-predict, adjustment for mean Kyte–Doolittle hydrophobicity is expected to reduce effect sizes. Indeed, several weaker amyloid enrichments attenuate after adjustment (e.g., lysosome, late endosome, lysosomal membrane, and metalloendopeptidase activity), whereas the strongest enrichments remain significant. Thus, while composition contributes, category-level enrichments are not explained solely by mean hydrophobicity or IDR length. Additional covariates such as predicted proximity to transmembrane segments or alternative IDR definitions may further refine membrane-associated categories; we treat such extensions as future work.

Figures

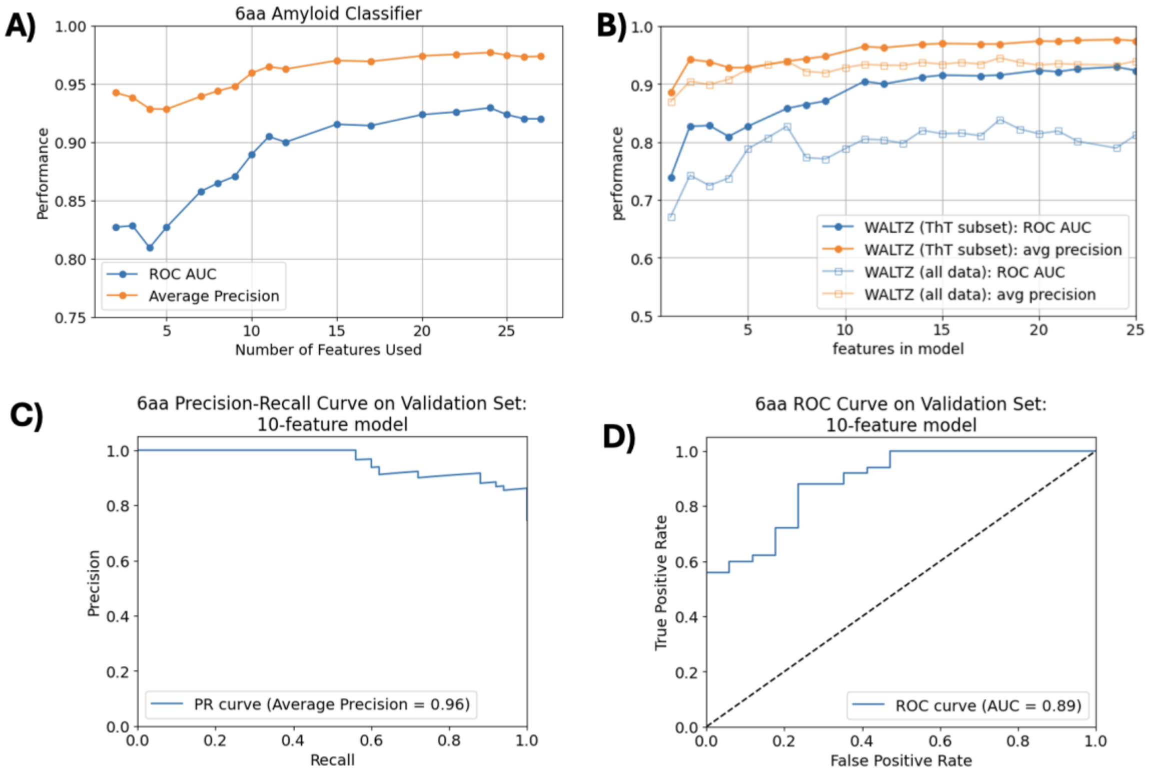

Fig. S1. A) Comparing ROC AUC and average precision as a function of the number of features selected with L1 regularization. B) Evaluating how adding more data, (i.e. the rest of the WALTZ data instead of just the WALTZ data with Th-T collected), hurts performance on the same validation set. C) PR curve and (D) ROC for the FETA model.

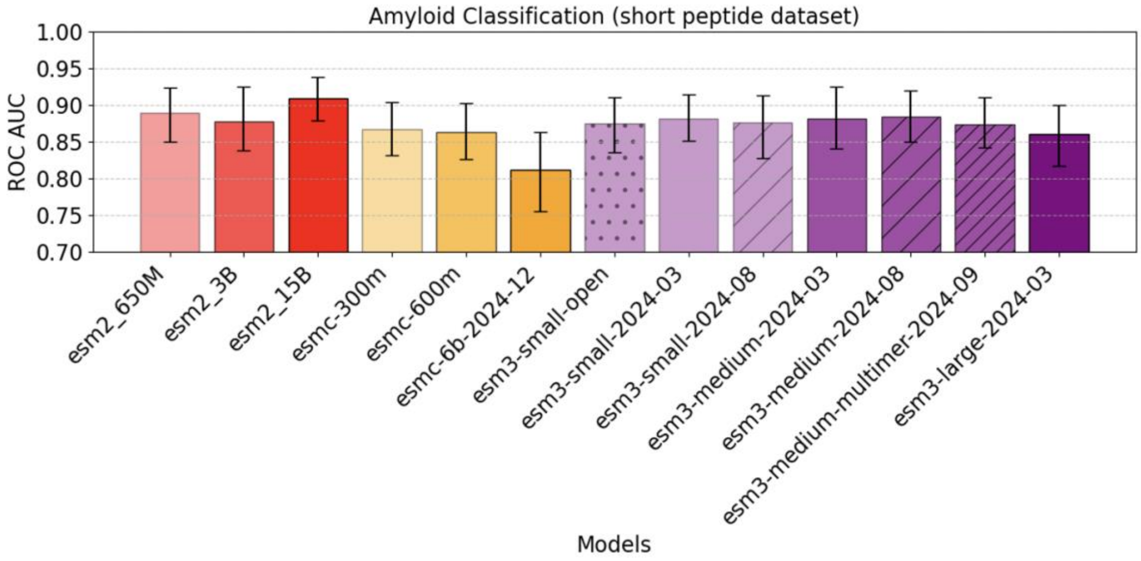

Fig. S2. Comparing pLMs' classification performance for the 6aa dataset. Error bars represent the 16th and 84th percentiles from 500 random train-test splits.

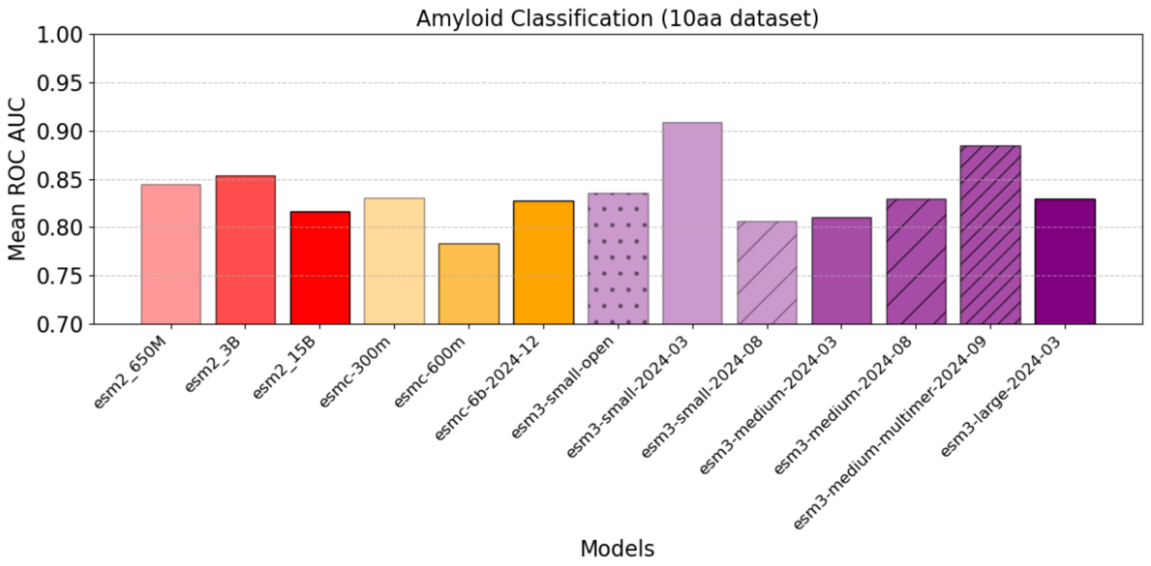

Fig. S3. Comparing pLMs' classification performance for the 10aa dataset, with LOOCV.

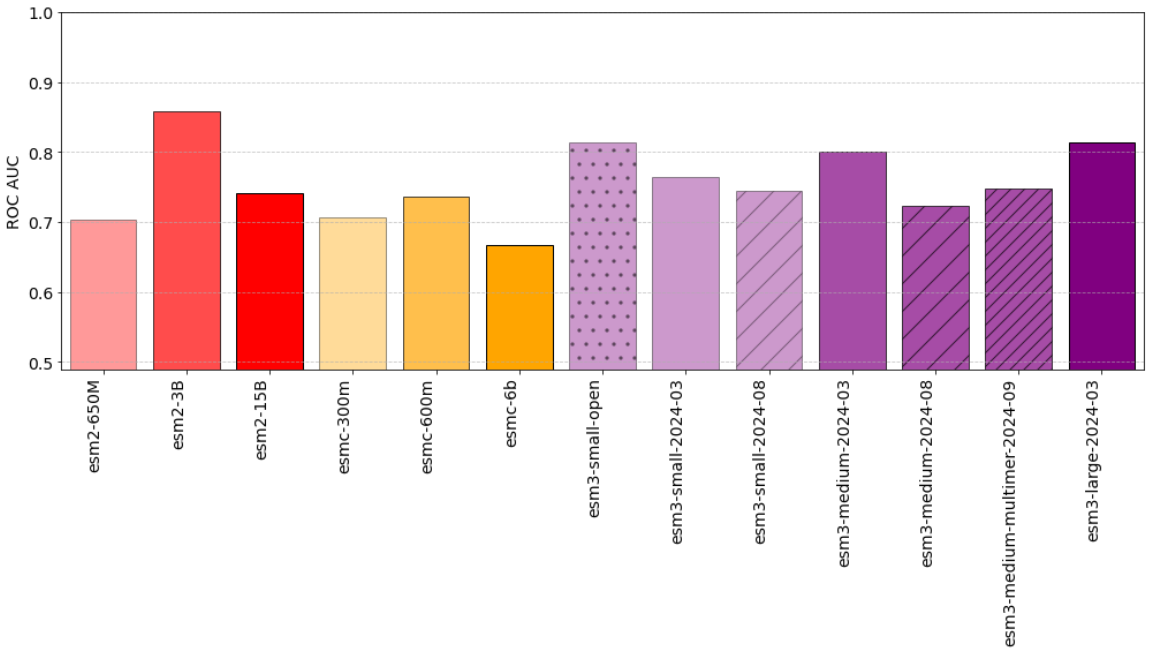

Fig. S4. Comparing pLMs' classification performance for the 15aa dataset, with LOOCV.

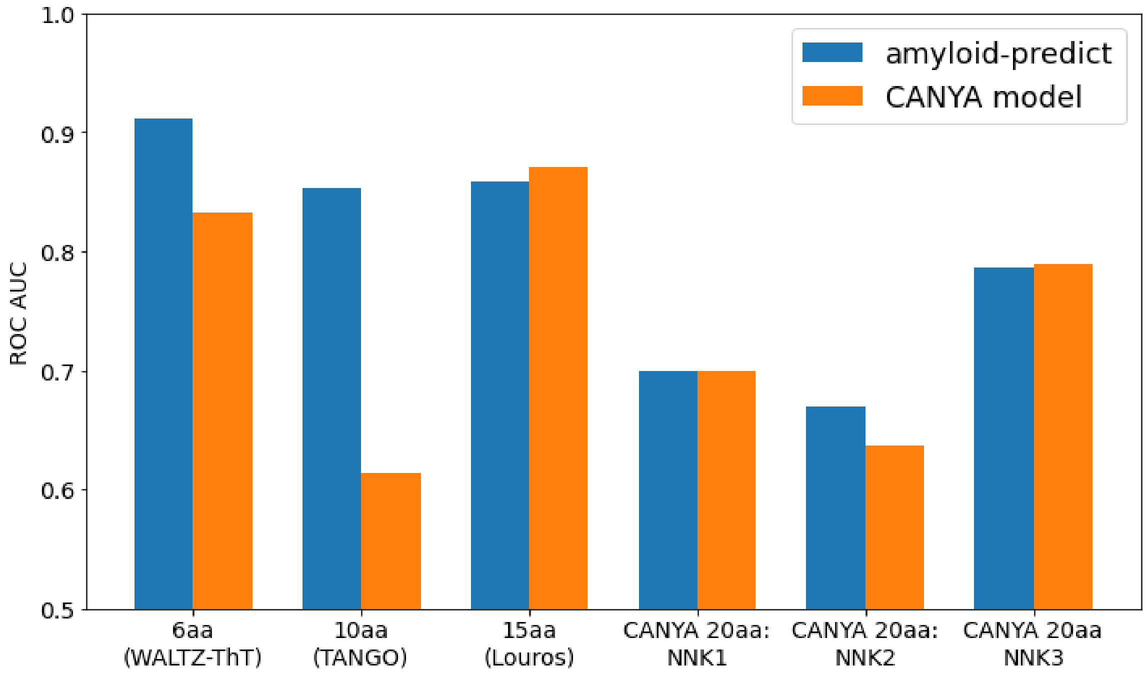

Fig. S5. Comparing CANYA performance to logistic regression models using ESM2-3B weights (i.e. amyloid-predict). For each amyloid-predict bar shown here, no feature selection or regularization tuning was done on the classifier; performance increases slightly with feature selection & regularization tuning for the 6aa dataset as discussed in the main text. The 6aa dataset performance was determined by training 500 models with random train-test split and we report the median ROC AUC. We trained additional amyloid-predict models on just the 20aa peptides in the CANYA authors' NNK1 (n=1913), NNK2 (n=1975), and NNK3 (n=2075) datasets, and the ROC AUC is similar to CANYA's scores. CANYA's inference is cheaper than that of amyloid-predict and its model is not pre-trained on evolutionary data like amyloid-predict.

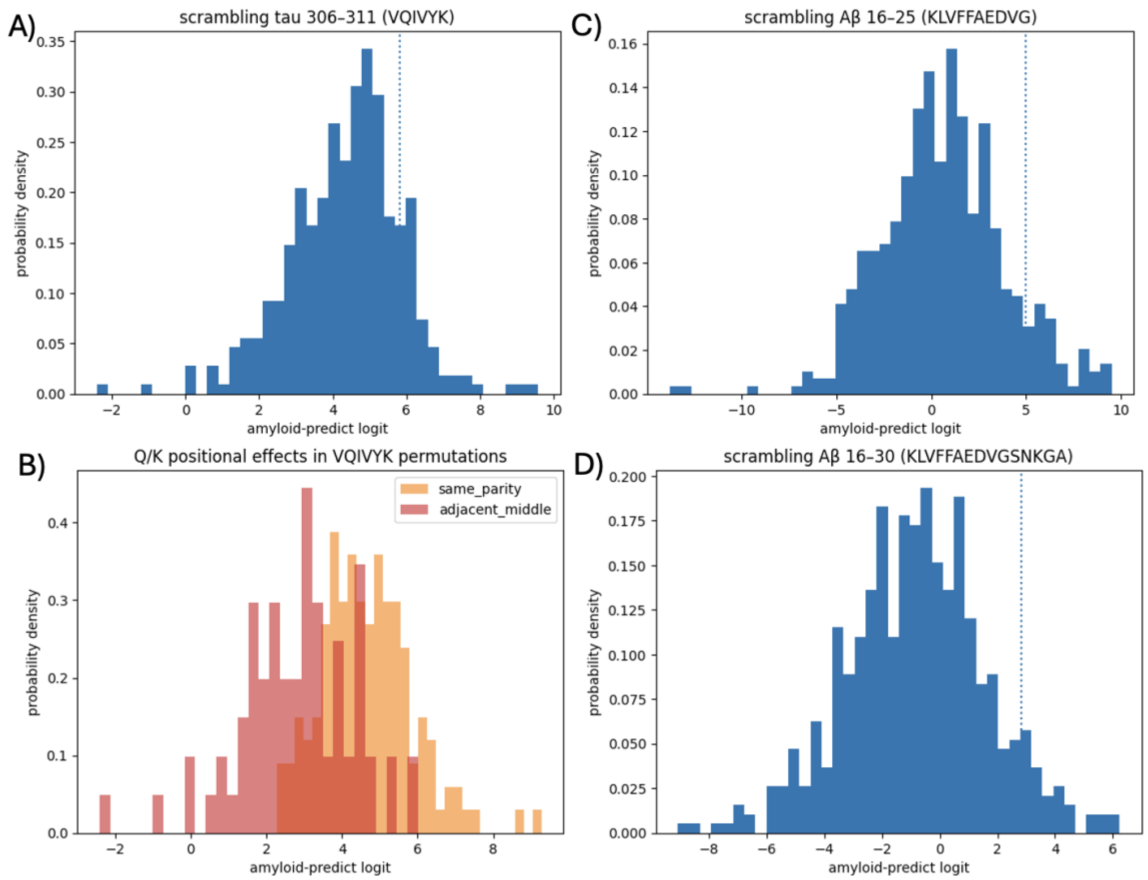

Fig. S6. Composition-preserving sequence scrambling reveals sensitivity of amyloid-predict to residue patterning. A) Distribution of amyloid-predict logit scores for all permutations of the tau PHF6 amyloidogenic hexapeptide (tau residues 306–311; VQIVYK). The vertical dashed line indicates the score of the native sequence. B) Subclassification of VQIVYK permutations based on the relative positioning of the charged residues Q and K. Permutations where Q and K occur at the same positional parity (both even or both odd residue ids; orange) tend to maintain higher predicted amyloid propensity, while sequences where Q and K are adjacent in the central region of the hexapeptide (red) show reduced scores, consistent with disruption of β-strand patterning. (C–D) Analogous composition-preserving scrambling analyses for longer Aβ amyloidogenic segments: Aβ16–25 (KLVFFAEDVG; C) and Aβ16–30 (KLVFFAEDVGSNKGA; D). Together, these controls demonstrate that amyloid-predict captures sequence patterning and contextual residue arrangement beyond amino-acid composition alone, with hydrophobic/hydrophilic spacing and clustering influencing predicted aggregation propensity.

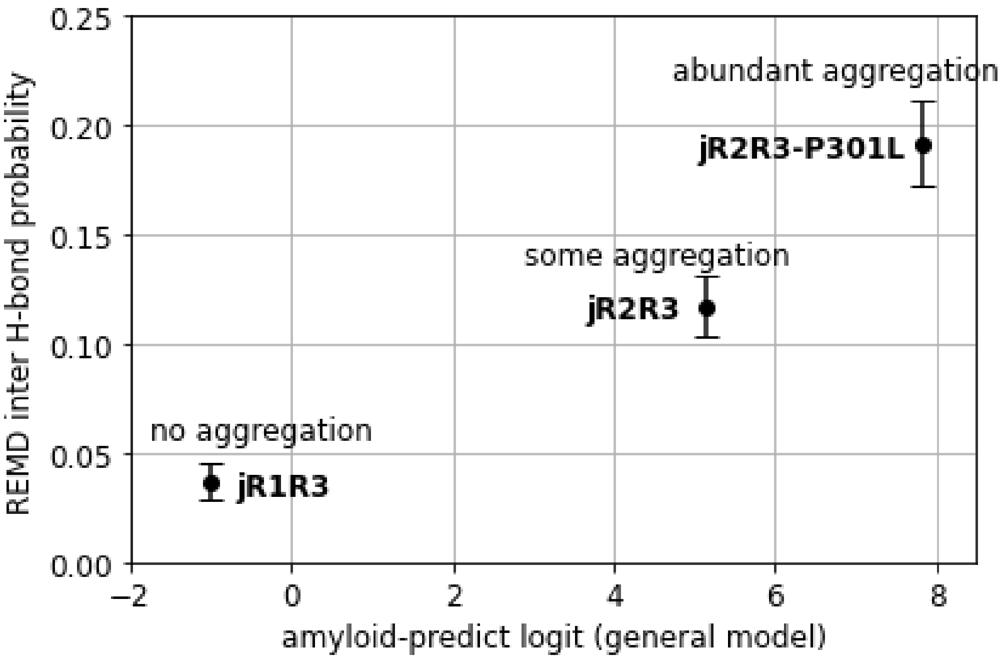

Fig. S7. The general amyloid classifier of amyloid-predict has logit scores that correlate with intermolecular H-bond probabilities from an ensemble in dimers, sampled by replica exchange molecular dynamics (REMD) in Vigers, et. al. (8) There is a single mutation between jR2R3-P301L and jR2R3, and there are four additional mutations to jR1R3. Experimentally observed aggregation behavior (from TEM and Th-T assay) are annotated above each point.

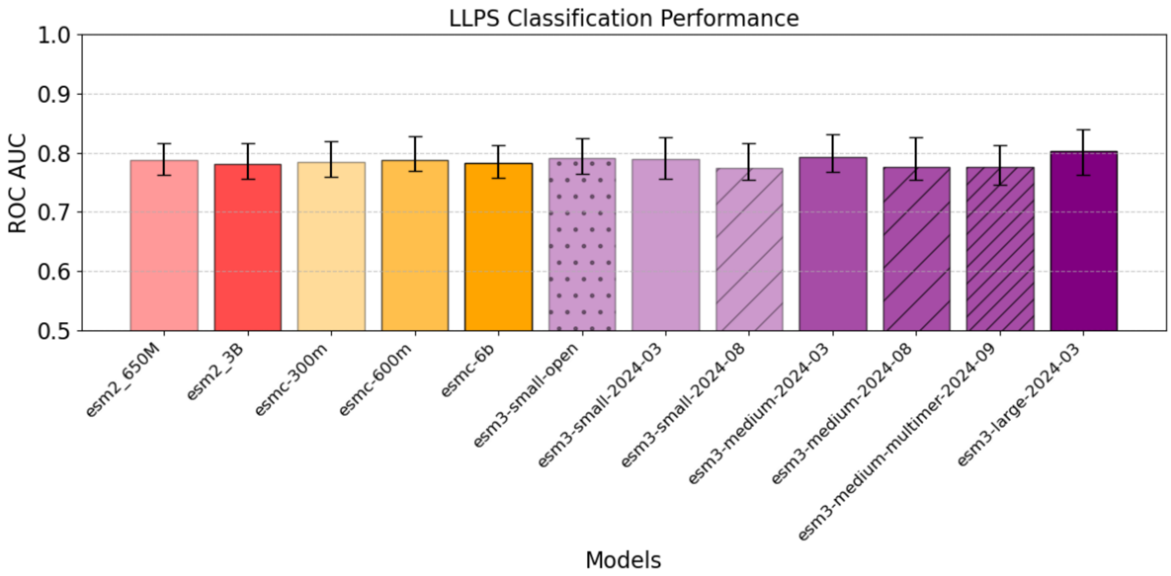

Fig. S8. Comparing pLMs' classification performance on the IDRome LLPS enrichment dataset. Models were trained with feature selection and regularization tuning as described in Appendix S1. 30 models were trained for each pLM with random train-test splits; the bars represent the median ROC AUC, while the error bars represent the 16th and 84th percentiles.

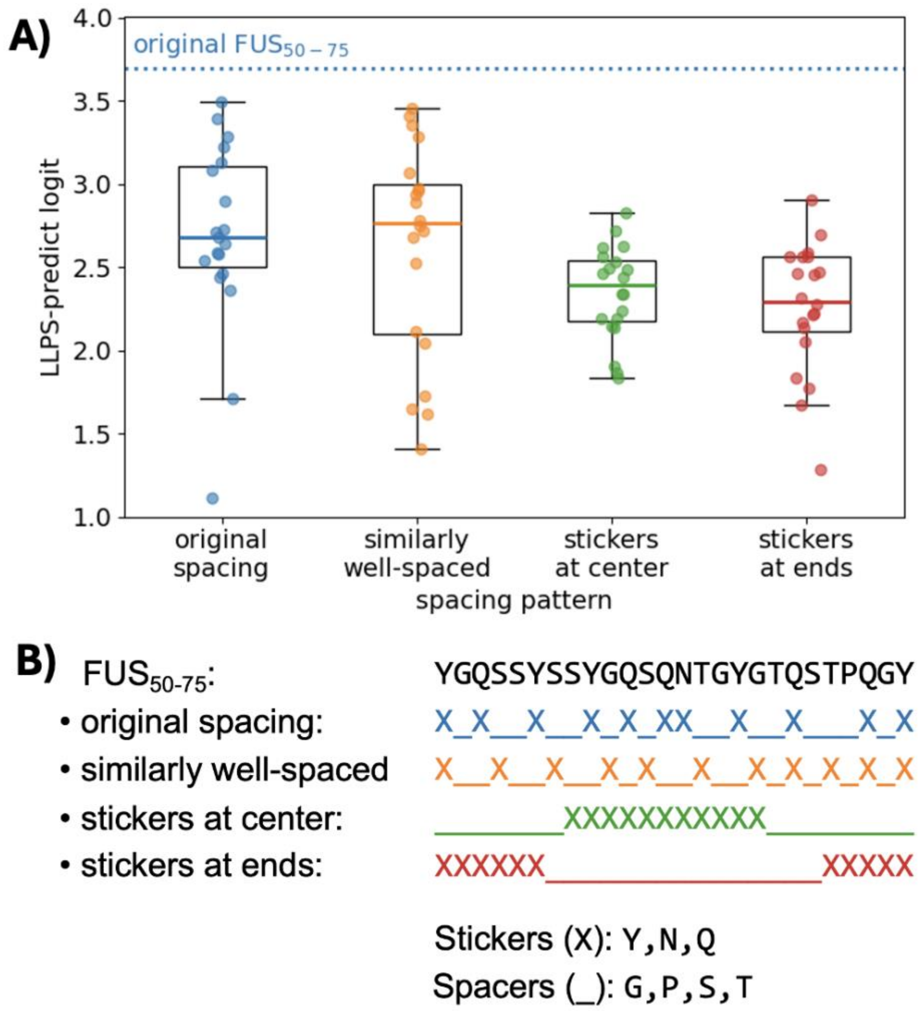

Fig. S9. Patterning sensitivity of LLPS-predict on a FUS low-complexity segment. A 26-residue segment of the FUS low-complexity domain (residues 50–75) was used to generate four classes of composition-matched sequences with identical numbers of "sticker" residues (Y, Q, N) and "spacer" residues (G, P, S, T), but different spatial patterning: (i) the original FUS pattern, (ii) a similarly well-spaced sticker arrangement, (iii) stickers clustered at the center, and (iv) stickers clustered at the ends. For each pattern class, 20 sequences were generated by shuffling residues subject to the corresponding spacing constraint. LLPS-predict scores (as logits) are shown as boxplots with individual sequences overlaid. The dotted line indicates the score of the original FUS$_{50-75}$ segment. LLPS-predict tends to assign higher scores to well-spaced sticker arrangements compared to clustered arrangements, demonstrating sensitivity to sequence patterning beyond amino acid composition. Stickers and spacers have been described previously in the literature (9) where stickers form reversible multivalent interactions; here we consider both aromatic and amide side chains to be stickers with amides capable of interacting multivalently via its hydrogen bond donor and acceptor.

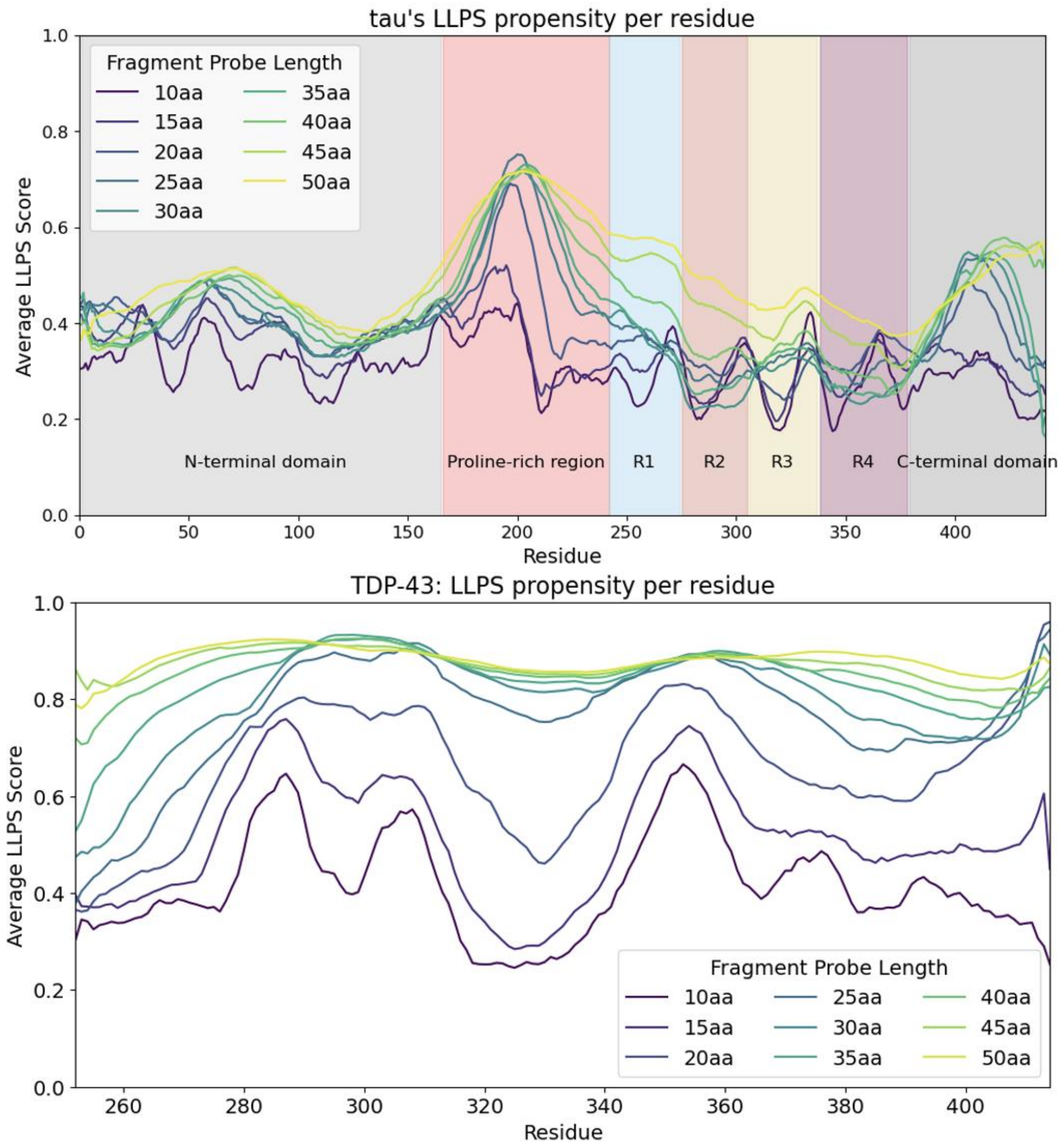

Fig. S10. Evaluating how the fragment probe length affects per-residue LLPS scores for tau (top) and TDP-43 (bottom). Shorter fragment probes are more sensitive and longer probes have more context and are aware of longer-ranged effects. We picked probe lengths of 15, 25, and 40 when analyzing the proteome, to include both the sensitive, local patterns and the long-ranged patterns.

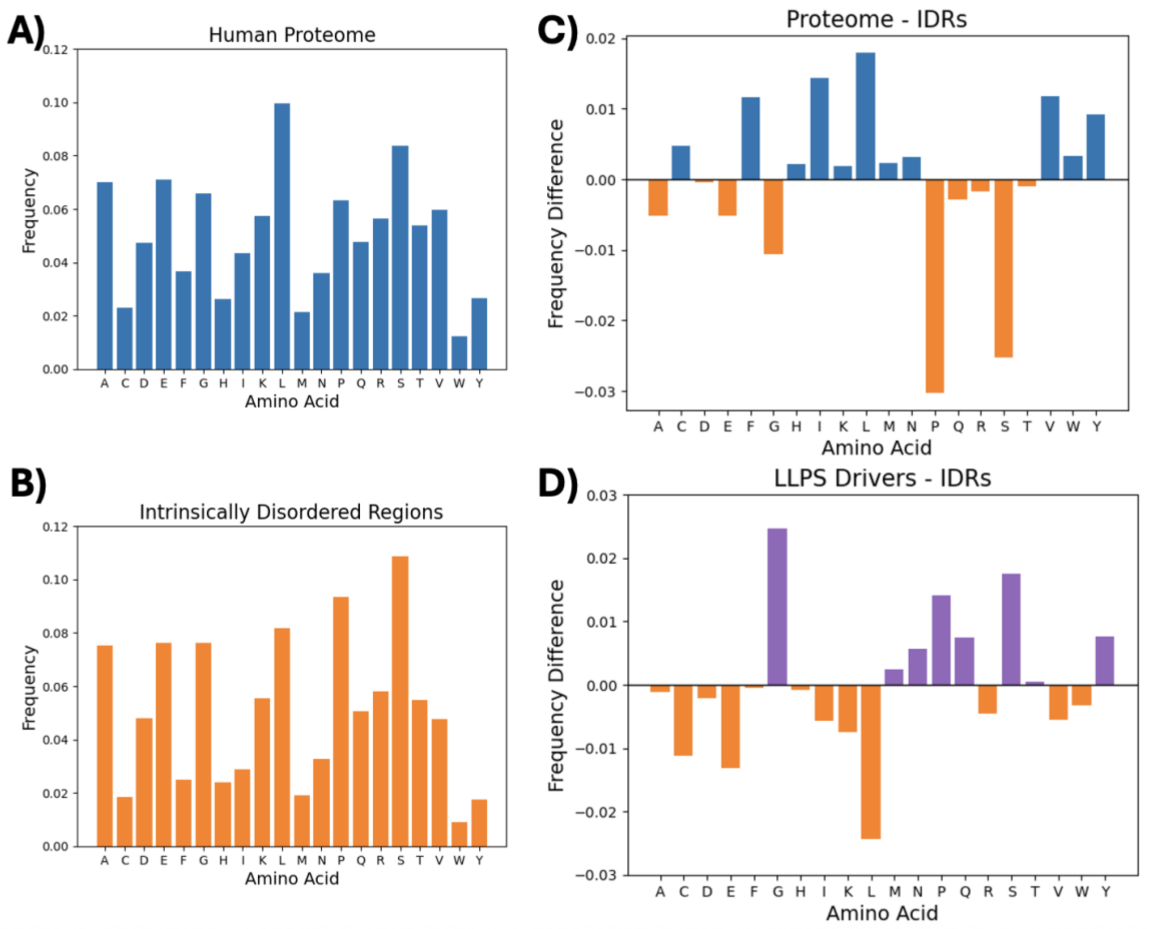

Fig. S11. Frequency of each amino acid in the proteome (A) and the IDRome (B). C) Difference between IDRome and proteome amino acid frequencies. The IDRome has more P's, S's, and G's. D) Difference between LLPS drivers identified by CD-CODE and the IDRome. LLPS drivers have more G's, S's, and P's than the rest of the IDRome.

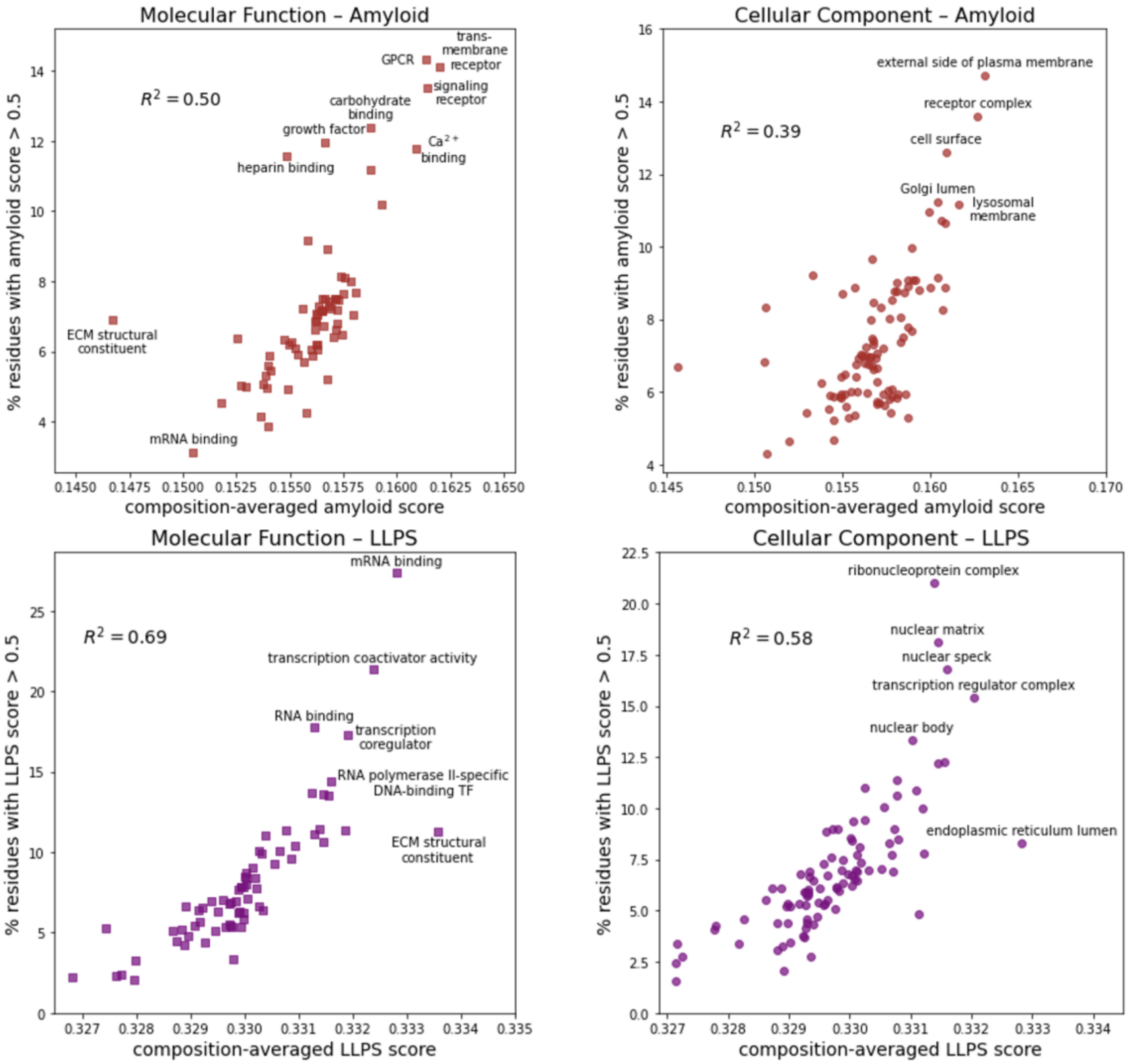

Fig. S12. Comparing the composition-averaged scores with the % residues with scores above 0.5. We show this for amyloid scores (top) and LLPS scores (bottom) for molecular function (left) and cellular localization (right) gene ontology categories. Composition-averaged scores are derived by multiplying the amino acid frequencies by the average score for that amino acid across the IDRome. We note the R^2 of the two axes in the top left of each plot.

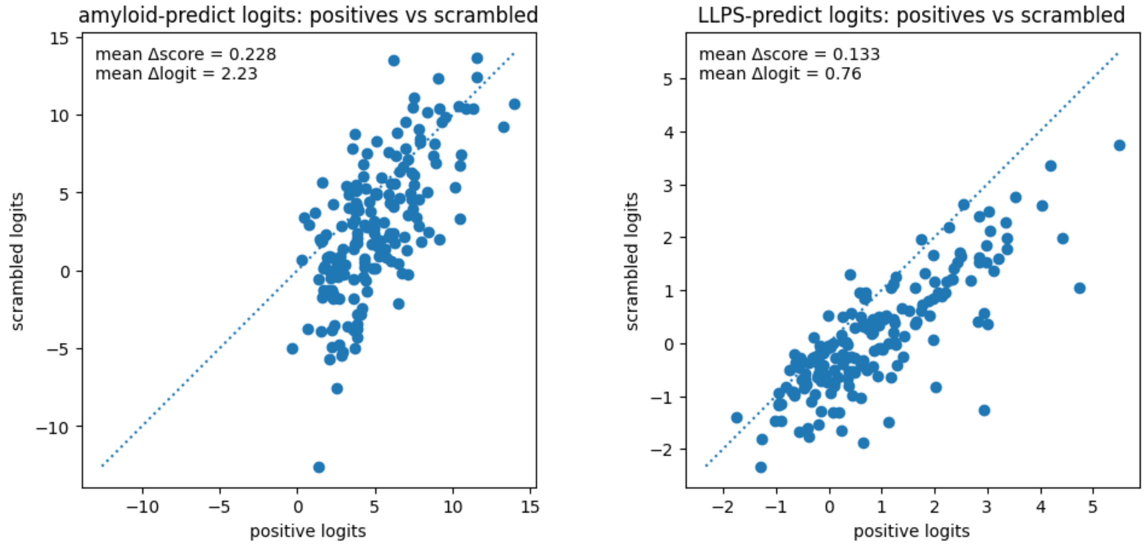

Fig. S13. Scrambling controls reveal distinct sensitivities to sequence patterning in amyloid-predict vs LLPS-predict. Each point compares the model logit for a positively labeled sequence (x-axis) to a composition-preserving scrambled version of the same sequence (y-axis); the dotted line indicates equality. Scrambling causes a larger mean decrease in amyloid-predict output (mean Δscore = 0.228; mean Δlogit = 2.23) than in LLPS-predict (mean Δscore = 0.133; mean Δlogit = 0.76), consistent with stronger dependence on residue patterning for amyloid-predict and comparatively greater composition dependence for LLPS-predict. Notably, this difference is observed despite LLPS-positive sequences being substantially longer on average, for which scrambling is more radical.

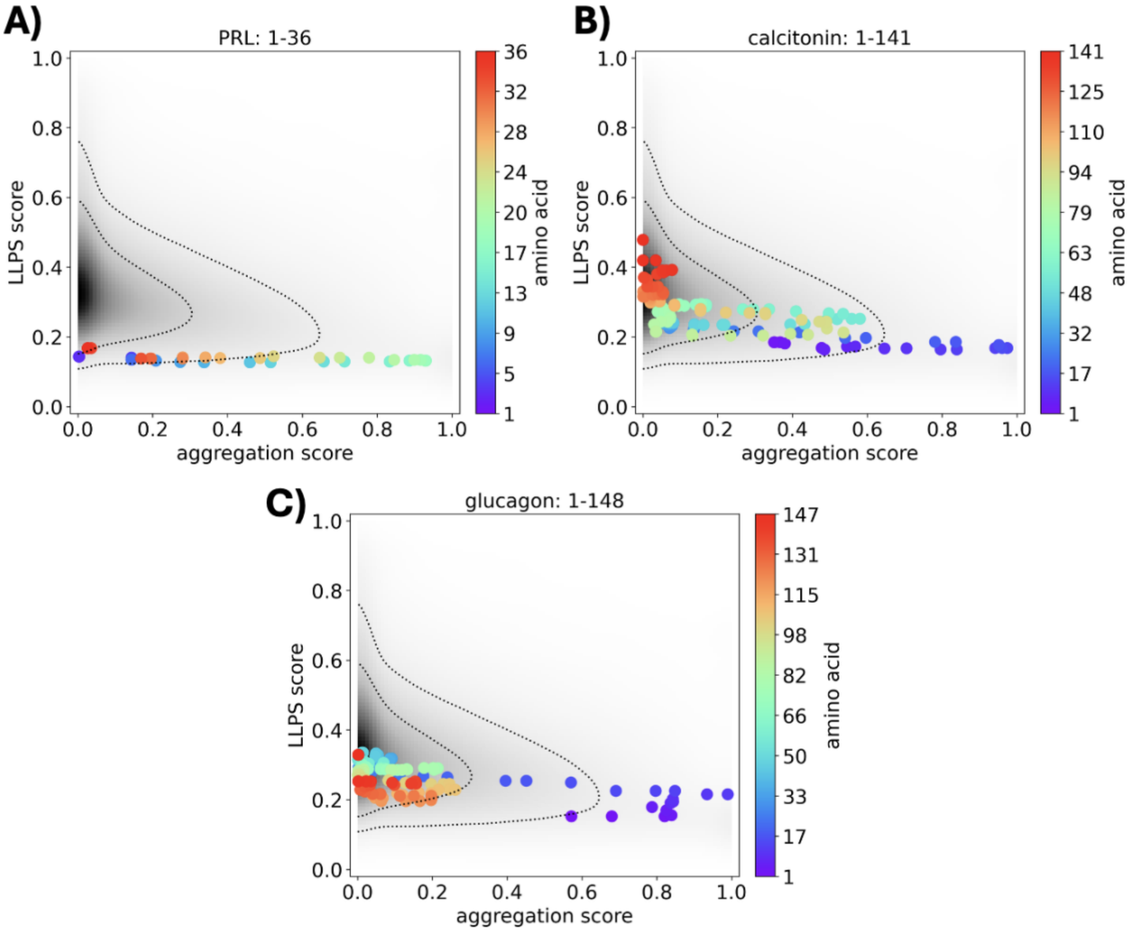

Fig. S14. 2D per-residue aggregation and LLPS scores of hormones prolactin (A), calcitonin (B), and glucagon (C). Color represents residue ID. Grays represent the histogram of scores for the rest of IDRome residues. Dotted line represent the 68th percentile and 90th percentile of residues in the IDRome. Each has regions with high aggregation scores.

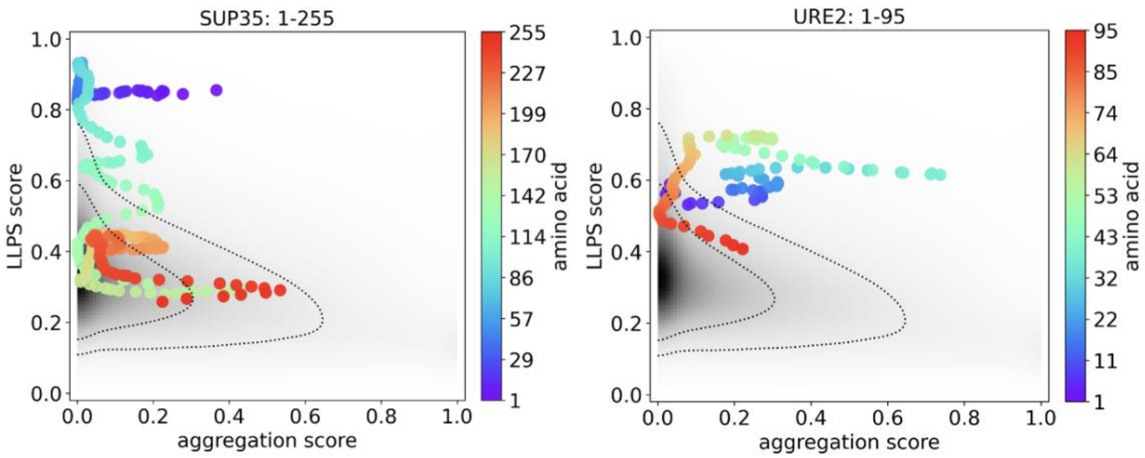

Fig. S15. 2D per-residue aggregation and LLPS scores of various known prionic yeast proteins: SUP35 (left) and URE2 (right). Each has regions of high LLPS scores and high amyloid aggregation scores.

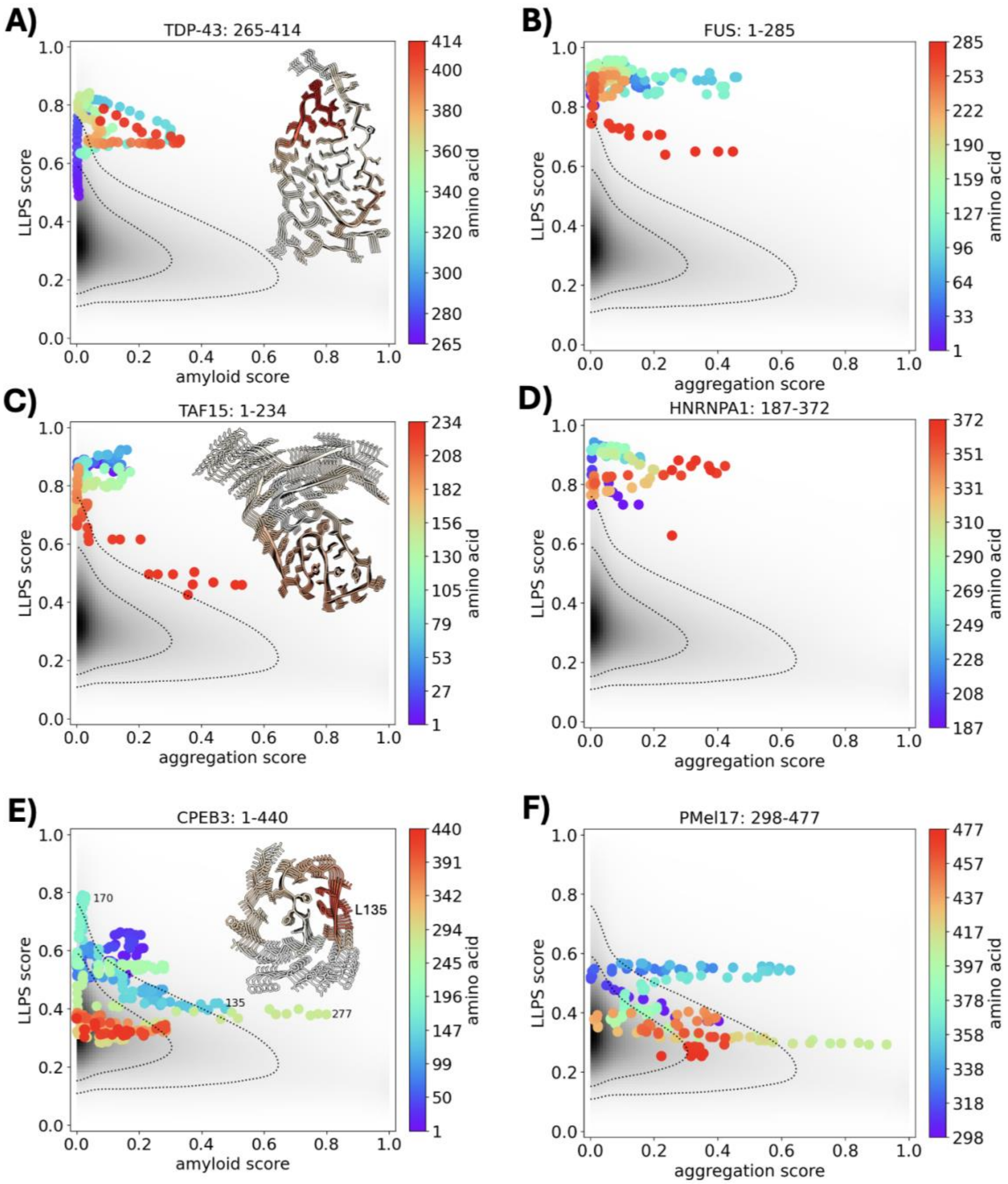

Fig. S16. 2D per-residue aggregation and LLPS scores of various known amyloid-forming proteins: TDP-43 (A), FUS (B), TAF15 (C), HNRNPA1 (D), CPEB3 (E), and PMel17 (F). (A) through (E) are RNA-associating proteins. (E) and (F) are known functional amyloids. Inset structures for TDP-43, TAF15, and CPEB3 are amyloid cryo-EM structures: #7PY2, #8ONS, #8SPA, respectively. Structures are colored by amyloid score using a white-to-red colormap with limits 0-0.3 for TDP-43 & TAF15, and limits 0.2-0.6 for CPEB3.

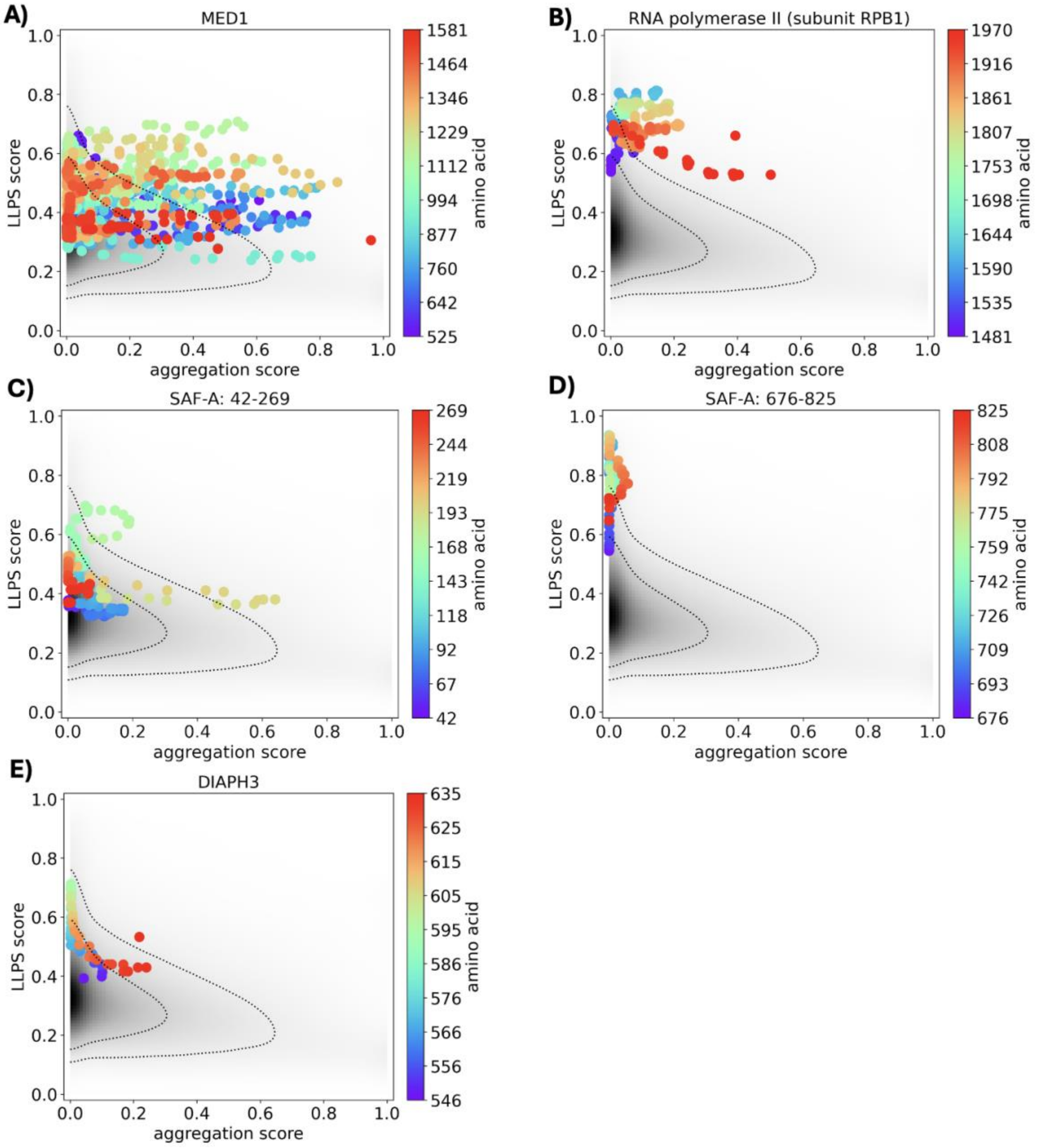

Fig. S17. 2D per-residue aggregation and LLPS scores from IDRs in MED1 (A), RNA polymerase II (B), SAF-A (C-D), and DIAPH3 (E).

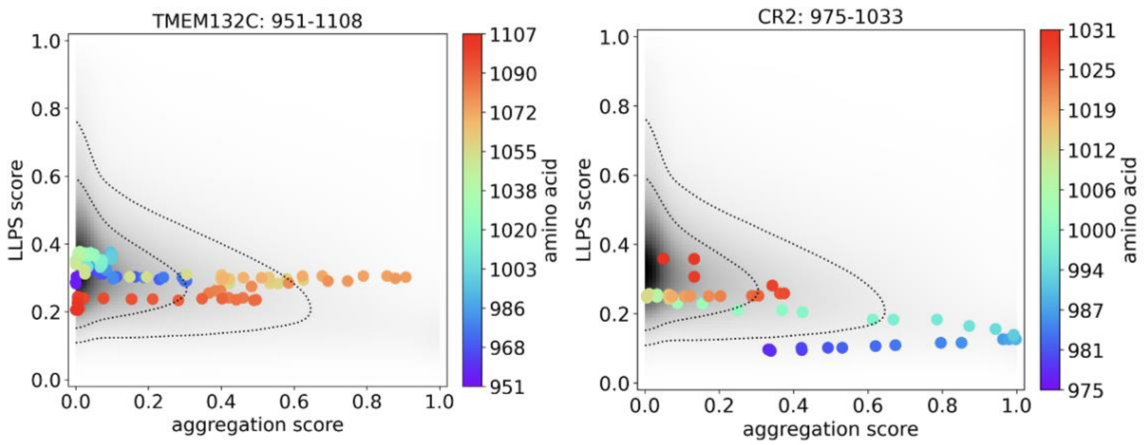

Fig. S18. 2D per-residue aggregation and LLPS scores of IDRs in TMEM132C (left) & CR2 (right). Both proteins are positively associated with Alzheimer's in recent GWAS (10) as mentioned in the Discussion section.

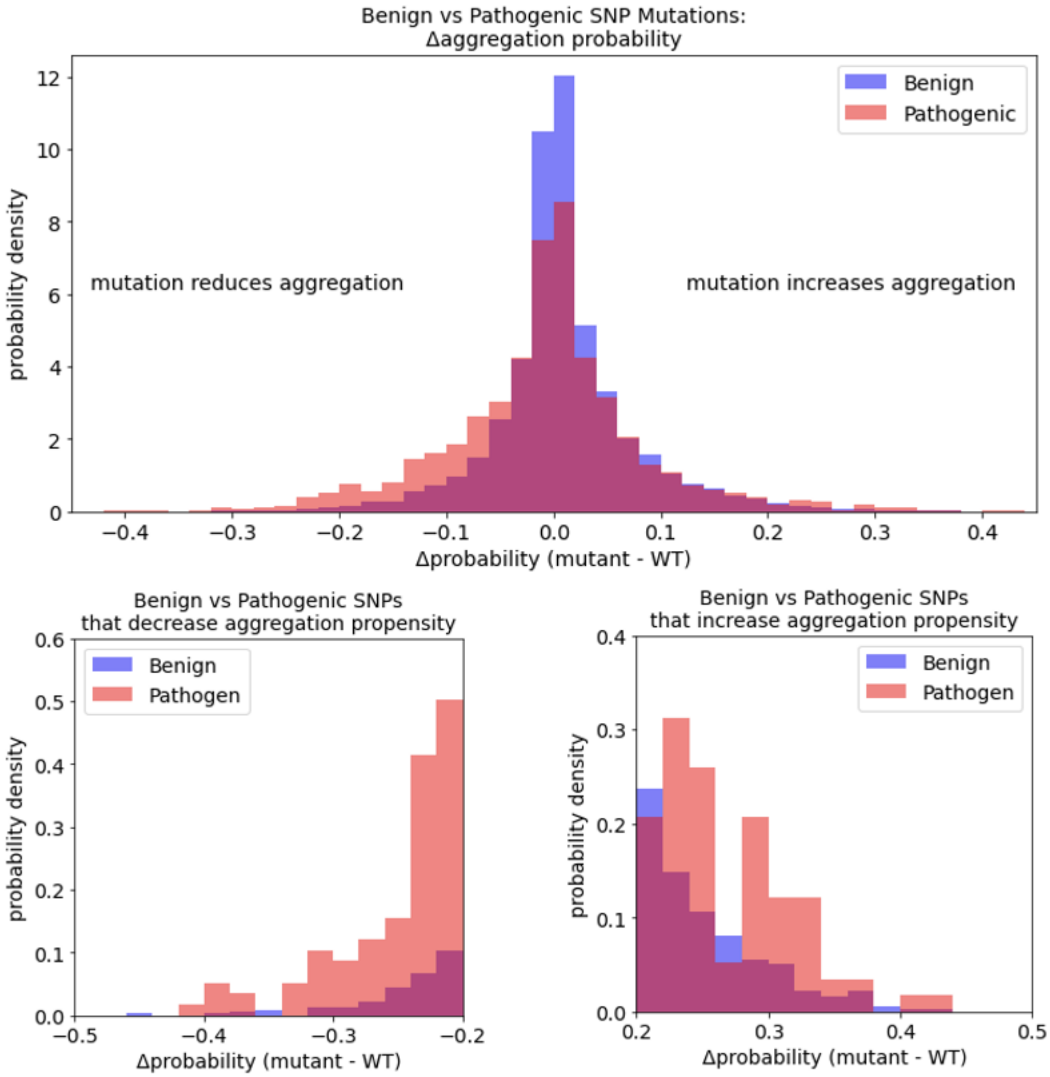

Fig. S19. SNP analysis of IDRome, histogramming the Δaggregation probability (relative to WT) at the site of the IDR mutation. Various pathogenic mutations induce particularly large increases and decreases in aggregation propensity, some of which we highlight in the main text. See Fig. S21 for description of how Δaggregation probabilities are calculated from the per-residue amyloid predict scores.

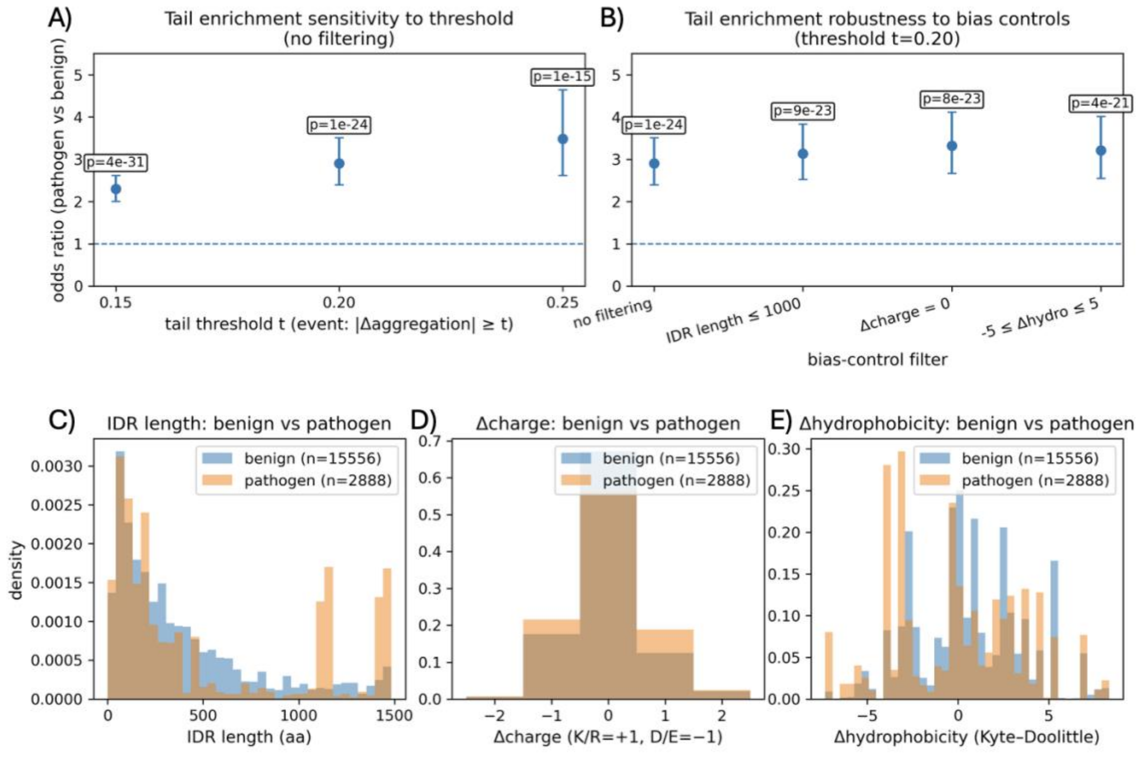

Fig. S20. Robust enrichment of large amyloid-propensity changes in pathogenic variants is not explained by simple compositional or structural biases. A) Odds ratio (pathogenic vs benign variants) for large amyloid-propensity changes (|Δaggregation| ≥ t) across increasing tail thresholds, showing consistent enrichment of pathogenic variants at more extreme changes. Error bars indicate 95% bootstrap confidence intervals; p-values are from Fisher's exact test. B) The enrichment remains robust after controlling for potential confounders, including intrinsically disordered region (IDR) length (≤1000 aa), net charge change (Δcharge = 0), and hydrophobicity change (−5 ≤ Δhydrophobicity ≤ 5). C–E) Distributions of IDR length, net charge change, and hydrophobicity change for benign vs pathogenic variants, respectively. While modest distributional differences exist, they do not appear to account for the observed tail enrichment of amyloid-propensity changes in pathogenic variants.

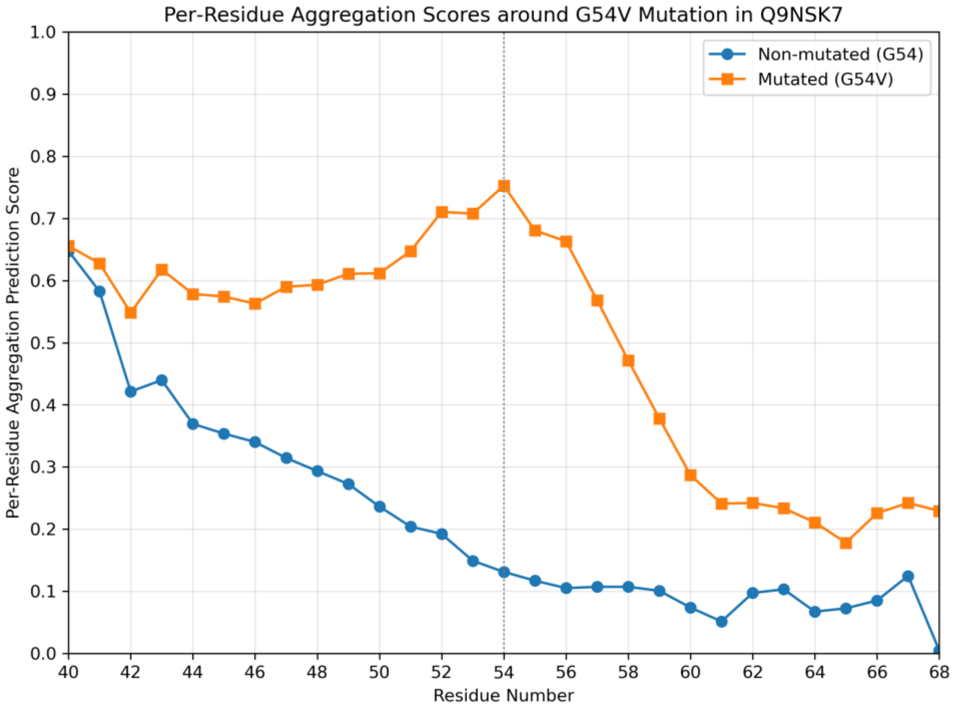

Fig. S21. Per-residue aggregation scores for G54V mutation and WT in C19orf12 (UnitProt ID Q9NSK7). These per-residue scores are aggregated into a Δprobability score that was used in Fig S19. Specifically, we subtract the average per-residue scores for the WT IDR from the mutant IDR to get the Δaggregation probability score.

SI References

1. N. Louros, *et al.*, Local structural preferences in shaping tau amyloid polymorphism. *Nat. Commun.* **15**, 1028 (2024).

2. A.-M. Fernandez-Escamilla, F. Rousseau, J. Schymkowitz, L. Serrano, Prediction of sequence-dependent and mutational effects on the aggregation of peptides and proteins. *Nat. Biotechnol.* **22**, 1302–1306 (2004).

3. M. Thompson, *et al.*, Massive experimental quantification allows interpretable deep learning of protein aggregation. *Sci. Adv.* **11**, eadt5111 (2025).

4. A. Rives, *et al.*, Biological structure and function emerge from scaling unsupervised learning to 250 million protein sequences. *Proc. Natl. Acad. Sci.* **118**, e2016239118 (2021).

5. G. Tesei, *et al.*, Conformational ensembles of the human intrinsically disordered proteome. *Nature* **626**, 897–904 (2024).

6. N. Rostam, *et al.*, CD-CODE: crowdsourcing condensate database and encyclopedia. *Nat. Methods* **20**, 673–676 (2023).

7. T. Hayes, *et al.*, Simulating 500 million years of evolution with a language model. *Science* **387**, 850–858 (2025).

8. M. P. Vigers, *et al.*, Water-directed pinning is key to tau prion formation. *Proc. Natl. Acad. Sci.* **122**, e2421391122 (2025).

9. J.-M. Choi, A. S. Holehouse, R. V. Pappu, Physical Principles Underlying the Complex Biology of Intracellular Phase Transitions. *Annu. Rev. Biophys.* **49**, 107–133 (2020).

10. D. Western, *et al.*, Proteogenomic analysis of human cerebrospinal fluid identifies neurologically relevant regulation and implicates causal proteins for Alzheimer’s disease. *Nat. Genet.* **56**, 2672–2684 (2024).
